# Group IV Phospholipase A_2_α Controls the Formation of Inter-Cisternal Continuities Involved in Intra-Golgi Transport

**DOI:** 10.1371/journal.pbio.1000194

**Published:** 2009-09-15

**Authors:** Enrica San Pietro, Mariagrazia Capestrano, Elena V. Polishchuk, Alessio DiPentima, Alvar Trucco, Pasquale Zizza, Stefania Mariggiò, Teodoro Pulvirenti, Michele Sallese, Stefano Tete, Alexander A. Mironov, Christina C. Leslie, Daniela Corda, Alberto Luini, Roman S. Polishchuk

**Affiliations:** 1 Department of Cell Biology and Oncology, Consorzio Mario Negri Sud, Chieti, Italy; 2 Department of Oral Sciences, University “G. D'Annunzio”, Chieti, Italy; 3 Department of Pediatrics, National Jewish Medical and Research Center, Denver, Colorado, United States of America; 4 Telethon Institute of Genetics and Medicine, Naples, Italy; Princeton University, United States of America

## Abstract

The organization of intra-Golgi trafficking and the nature of the transport intermediates involved (e.g., vesicles, tubules, or tubular continuities) remain incompletely understood. It was recently shown that successive cisternae in the Golgi stack are interconnected by membrane tubules that form during the arrival of transport carriers from the endoplasmic reticulum. Here, we examine the mechanisms of generation and the function of these tubules. In principle, tubule formation might depend on several protein- and/or lipid-based mechanisms. Among the latter, we have studied the phospholipase A_2_ (PLA_2_)-mediated generation of wedge-shaped lysolipids, with the resulting local positive membrane curvature. We show that the arrival of cargo at the Golgi complex induces the recruitment of Group IVA Ca^2+^-dependent, cytosolic PLA_2_ (cPLA_2_α) onto the Golgi complex itself, and that this cPLA_2_α is required for the formation of the traffic-dependent intercisternal tubules and for intra-Golgi transport. In contrast, silencing of cPLA_2_α has no inhibitory effects on peri-Golgi vesicles. These findings identify cPLA_2_α as the first component of the machinery that is responsible for the formation of intercisternal tubular continuities and support a role for these continuities in transport through the Golgi complex.

## Abstract 2

The enzyme phospholipase A2 (cPLA_2_α) is involved in the formation of intercisternal tubules that mediate transport of proteins within the Golgi complex.

## Introduction

After their synthesis in the endoplasmic reticulum (ER), cargo proteins move to the Golgi complex. This unique structure comprises numerous compact stacks of cisternae that are laterally interconnected into the Golgi “ribbon” through tubular-reticular networks (“non-compact zones” [1]). Cargo proteins then traverse the Golgi cisternal subcompartments (where they are glycosylated), and at the *trans*-Golgi face they are sorted and delivered to their further destinations via large tubular/pleiomorphic carriers [2]–[6].

Despite significant advances in recent years, important aspects of the organization of intra-Golgi trafficking remain unclear. Three main intra-Golgi transport models have been traditionally considered for higher eukaryotes: trafficking by anterograde vesicles [7], trafficking by compartment maturation-progression [8]–[11], and trafficking by diffusion via tubular continuities joining different Golgi cisternae [12]–[18]. Among these, the cisternal progression model has recently gained broad consensus, although the nature of the intermediates involved in this mechanism (vesicles or intercisternal tubules) remains unclear. In addition, more recently, a new model has been proposed by which cargo molecules mix rapidly throughout the stack (compatible with cargo diffusion via intercisternal continuities) and then partition into specialized export domains before leaving the Golgi complex [19].

Thus far, intra-Golgi transport models, the last, diffusion via tubular continuities, has received the least attention [12]–[17], and even the very existence of intercisternal tubules has long remained an issue of debate [20]. The main reasons for this uncertainty have probably been the technical difficulties of detecting the convoluted structures of the intercisternal tubules by traditional electron microscopy (EM) and the traffic-related dynamics of these tubules [18],[21]. Over the last few years, however, tomography studies have indicated that tubules can be shown to join successive Golgi cisternae in animal cells, and that they form specifically under conditions of active trafficking [17],[18],[21]. Thus, although intercisternal tubules have still not been completely characterized (for instance, quantitative data on their frequency remain scarce [18],[22]), they can be considered as potential players in intra-Golgi trafficking. It is therefore of interest to understand and manipulate their molecular mechanisms of formation.

The formation of an intercisternal tubule is likely to depend on several molecular events, including mechanical deformation by specialized proteins and changes in the distribution/geometry of the lipids within the membrane bilayer [23],[24]. These latter can be induced in several ways, including the formation of local spontaneous positive membrane curvature via the generation of lysolipids and fatty acids by PLA_2_ activity [24],[25]. Indeed, a role for PLA_2_ in membrane shaping has been suggested in vitro [26], as well as in a series of in vivo studies that have indicated that chemical blockers of PLA_2_ suppress tubule formation in several endomembrane compartments and inhibit the related trafficking steps (see Brown et al. [27] for review).

Here, we show that transport through the Golgi complex coincides with the rapid recruitment of a specific molecular PLA_2_ isoform onto Golgi membranes: Group IVA, Ca^2+^-dependent cytosolic PLA_2_ (cPLA_2_α). The activity of cPLA_2_α is required for the formation of intercisternal connections in the Golgi. In addition, we show that treatments that inhibit cPLA_2_α and suppress intercisternal tubule formation also block intra-Golgi trafficking of several cargo proteins. These data identify cPLA_2_α as a component of the machinery underlying intercisternal tubular continuities and support a role for these continuities in intra-Golgi transport.

## Results

As indicated above, PLA_2_ inhibitors have been reported to suppress membranous tubules that extend from different cellular compartments, including the Golgi complex [28],[29]. We thus sought to identify the Golgi-associated PLA_2_ isoform that might serve as the molecular target of these inhibitors and might be involved in the regulation of Golgi-associated tubular structures. The superfamily of PLA_2_ enzymes consists of 15 groups comprising secretory and cytosolic enzymes, with the latter divided into Ca^2+^-sensitive (cPLA_2_s, or Group IV PLA_2_s) and Ca^2+^-insensitive (Group VI, VII, and VIII PLA_2_s) isoforms [30]. Among those that are Ca^2+^ sensitive, one of the Group IV isoforms, cPLA_2_α, that is normally cytosolic has been reported to associate preferentially with the Golgi complex upon moderate increases in cytosolic Ca^2+^ concentrations [31]–[34]. This affinity for Golgi membranes prompted us to examine whether cPLA_2_α might itself be involved in intercisternal tubule formation and intra-Golgi trafficking.

### Traffic Induces Binding of cPLA_2_α to the Golgi Complex in a Ca^2+^-Dependent Fashion

**Figure 1:**
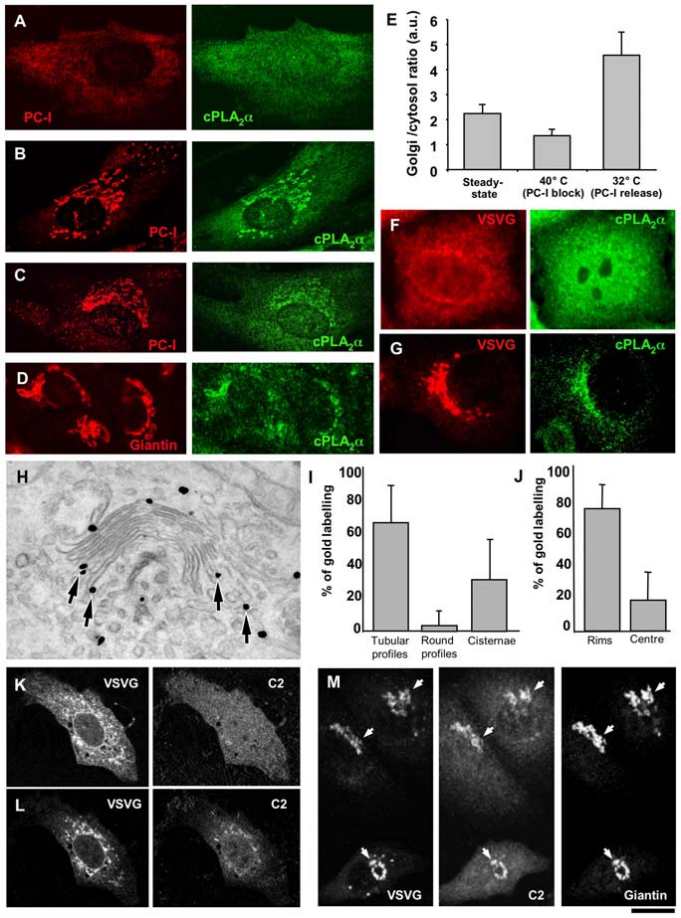
cPLA_2_α is recruited to Golgi membranes upon arrival of cargo from the ER. (A, B) HFs were incubated for 3 h at 40°C (A) and then shifted to 32°C for 30 min in the presence of ascorbic acid (B), or HFs (C) and HepG2 cells (D) were grown under steady-state conditions. After fixing, the cells were double labelled with antibodies against PC-I and cPLA_2_α and examined under the confocal microscope. (A) When PC-I was trapped in the ER after the 40°C block, cPLA_2_α enrichment was not detected in the Golgi area. (B) Arrival of PC-I at the Golgi complex after release of the temperature block induced cPLA_2_α binding to the Golgi complex. (C, D) Under steady-state conditions, the cells were fixed, labelled with antibodies against cPLA_2_α and either PC-I (C) or giantin (D). Confocal microscopy reveals that here some cPLA_2_α can be seen in the Golgi area together with PC-I (C) and giantin (D). (E) Fluorescence intensity of cPLA_2_α in the Golgi region (defined as giantin-positive area) was quantified and normalized to cPLA_2_α intensity in the cytosol of HFs. Plot shows that Golgi/cytosol ratio of cPLA_2_α (mean±SD; *n* = 20 cells) decreases in cells subjected to 40°C transport block, but increases over the levels detected at steady-state conditions upon block release (at 32°C). (F, G) HeLa cells were infected with VSV and kept at 40°C for 3 h to accumulate VSVG in the ER. The cells were then fixed (F) or shifted to 32°C for 30 min to allow VSVG to exit from the ER (G), and prepared for confocal microscopy. Immunofluorescence labelling shows a diffuse pattern of cPLA_2_α when VSVG is blocked in the ER (F). In contrast, cPLA_2_α undergoes recruitment to the Golgi membranes as soon as VSVG arrives at the Golgi complex from the ER (G). (H) HeLa cells were transfected with cDNA encoding full length cPLA_2_α fused with GFP (cPLA_2_α-GFP), infected with VSVG, and subjected to the 40°C block. The cells were then fixed 30 min after the 40°C block release, to allow VSVG to reach the Golgi complex, and processed for immuno-gold EM with an anti-GFP ab, to reveal cPLA_2_α-GFP localization. After activation of VSVG transport, a cPLA_2_α-GFP signal was detected at the rims of the Golgi cisternae and flanking tubular structures (arrows). (I, J) Quantification of gold labelling (mean±SD; *n* = 30 stacks) at the Golgi complex (see Materials and Methods) shows most of the cPLA_2_α-GFP is bound to the tubular profiles (I) and the rims of the cisternae (J). (K, L) HeLa cells were co-transfected with the cDNAs encoding VSVG-YFP and the C2 domain of cPLA_2_α fused with GFP (C2-GFP), kept at 40°C for 3 h (K), and observed in vivo under the confocal microscope during VSVG-YFP release from the ER. Images extracted from the time-lapse sequence show that C2-GFP moves from the cytosol (K) to the Golgi membranes (L) when VSVG appears within the Golgi area. (M) HeLa cells were transfected and incubated as above (K, L), and then observed under the confocal microscope and fixed during VSVG release when detectable amounts of C2-GFP started to appear in the perinuclear area. Further staining with anti-giantin antibodies reveals overlap of C2-GFP perinuclear signal with giantin labelling (arrows). Scale bar, 7 µm (A–D, F, G), 160 nm (H), 9 µm (K–M).

Initially, we asked whether cPLA_2_α is recruited to the Golgi complex during activation of transport through this organelle. We first generated a procollagen-I (PC-I) traffic pulse in human fibroblasts (HFs) using the 40–32°C transport synchronization protocol [8],[35]. During the 40°C block, PC-I was diffusely distributed in the ER and cPLA_2_α was mostly cytosolic (Figure 1A). After releasing the block, PC-I reached the Golgi complex, where its levels continued to increase for 30 min (Figure 1B). At the same time, remarkably, cPLA_2_α was partially recruited to the Golgi complex (Figure 1B). This cPLA_2_α increase on the Golgi complex lasted for at least 30 min. Similar experiments were carried out using the same 40–32°C transport synchronization protocol [35] in other cell types (HeLa and MDCK cells) with the temperature sensitive mutant of the vesicular somatitis virus glycoprotein (VSVG) as cargo, a well-characterized trafficking marker [35],[36]. VSVG can be expressed by either transfecting or infecting cells with vesicular somatitis virus (VSV). In both cases, the generation of a traffic pulse induced the recruitment of cPLA_2_α to the Golgi complex (Figure 1F and 1G), as seen with PC-I. We further examined the distribution of cPLA_2_α in the Golgi by immuno-EM. We fixed VSV-infected cells expressing cPLA_2_α-GFP both during the 40°C block and after releasing the block, and labelled the cells with an anti-GFP antibody (ab). Most of the cPLA_2_α-GFP was dispersed in the cytosol during the 40°C traffic block, with very few gold particles on the ER and the Golgi complex. In contrast, the cells fixed 30 min after releasing the block (i.e., during a traffic wave) showed significant amounts of cPLA_2_α-GFP at the rims of the Golgi cisternae and on rim-associated tubules (Figure 1H–1J). Some cPLA_2_α-GFP labelling was still seen in the cytosol and at low levels in the ER, but not on other intracellular membranes. Notably, this distribution is consistent with an action of cPLA_2_α in the formation of tubules from cisternal rims (see below).

To verify that this cPLA_2_α recruitment to the Golgi complex was due solely to changes in membrane transport rather than to the temperature changes required for inducing and releasing the trafficking block, we infected HeLa cells with VSV (as above) and then treated cells for 2 h with cycloheximide at 40°C (to deplete the cells of cargo and thus reduce transport [18]). Under these conditions, the temperature shift from 40°C to 32°C did not induce any significant changes in cPLA_2_α localization (unpublished data).

We next investigated whether recruitment of cPLA_2_α to the Golgi complex could be seen also under physiological steady-state trafficking conditions, rather than only during traffic waves. Indeed, HFs showed detectable levels of cPLA_2_α recruitment to the Golgi complex also at steady-state (Figure 1C). Moreover, when PC-I transport was inhibited using the 40°C temperature block, cPLA_2_α lost its association with the Golgi complex, and when trafficking resumed at 32°C, cPLA_2_α “rebounded” to high levels on the Golgi complex (Figure 1A and 1B). Quantification of cPLA_2_α levels on the Golgi under these conditions is shown in Figure 1E. Recruitment of cPLA_2_α to the Golgi at steady-state was detected also in other cell types (HeLa, MDCK, and HepG2). In particular, in liver hepatoma HepG2 cells, which are professional secretor cells that release up to 20 different soluble serum proteins [37], the Golgi showed relatively high PLA_2_ levels (Figure 1D). Thus, collectively, these data indicate that both “pulsed” and steady-state trafficking through the Golgi complex induces the association of cPLA_2_α with the Golgi membranes.

What is the mechanism of cPLA_2_α recruitment to the Golgi complex? The binding of cPLA_2_α to membranes has been shown to be due both to the Ca^2+^-binding C2 domain and to the catalytic domain of this protein. The C2 domain is required for initiating membrane association, in a process that depends exclusively on the intracellular Ca^2+^ concentration ([Ca^2+^]_i_) and has thus been proposed to function as a calcium sensor [32], while the catalytic domain prolongs this membrane binding of cPLA_2_α even after the [Ca^2+^]_i_ has returned to lower levels [32]. We monitored the in vivo dynamics of the calcium-sensor C2 domain of cPLA_2_α fused with GFP (C2-GFP [32]) during the 40–32°C traffic pulse. Figure 1K shows that C2-GFP had a diffuse cytosolic pattern when most of the cargo protein (VSVG-YFP) was arrested in the ER. However, as soon as VSVG started to concentrate within the Golgi complex after the release of the traffic block (after 150–250 s), a significant portion of C2-GFP moved from the cytosol to the Golgi complex (Figure 1L and 1M; Video S1), and then gradually returned to the cytosol. Under the same conditions, the full-length cPLA_2_α protein shifted to the Golgi complex and remained there for over 30 min (see above). This behaviour of C2-GFP and cPLA_2_α-GFP suggests that the trafficking induces a transient increase in [Ca^2+^]_i_, which in turn initiates cPLA_2_α recruitment to the Golgi complex [32], an interaction that would then be prolonged by the catalytic portion of cPLA_2_α. While this increase remains to be further defined and clarified, it could be related to the high lumenal Ca^2+^ concentrations in the Golgi complex [38] and the localization in the Golgi area of the signalling machinery that is involved in the release of Ca^2+^ from intracellular stores [38],[39]. Thus, the specific recruitment of cPLA_2_α to the Golgi complex can be explained by a local traffic-induced Ca^2+^ release from the Golgi complex and probably, additionally, by an intrinsic affinity of cPLA_2_α for the Golgi (possibly due to its affinity for lipids and proteins that are enriched in Golgi membranes [40]–[42]). Potentially related to this, Ca^2+^ released from the Golgi cisternae has been shown to be required for intra-Golgi transport [43].

In summary, traffic moving through the Golgi complex triggers the recruitment of cPLA_2_α to the rims of the Golgi cisternae, possibly through a signalling mechanism [44] that might induce the local Ca^2+^ increase that is required for cPLA_2_α binding to membranes [32].

### Inhibiting cPLA_2_α Reduces Inter-Cisternal Tubules and Suppresses Intra-Golgi Transport

**Figure 2:**
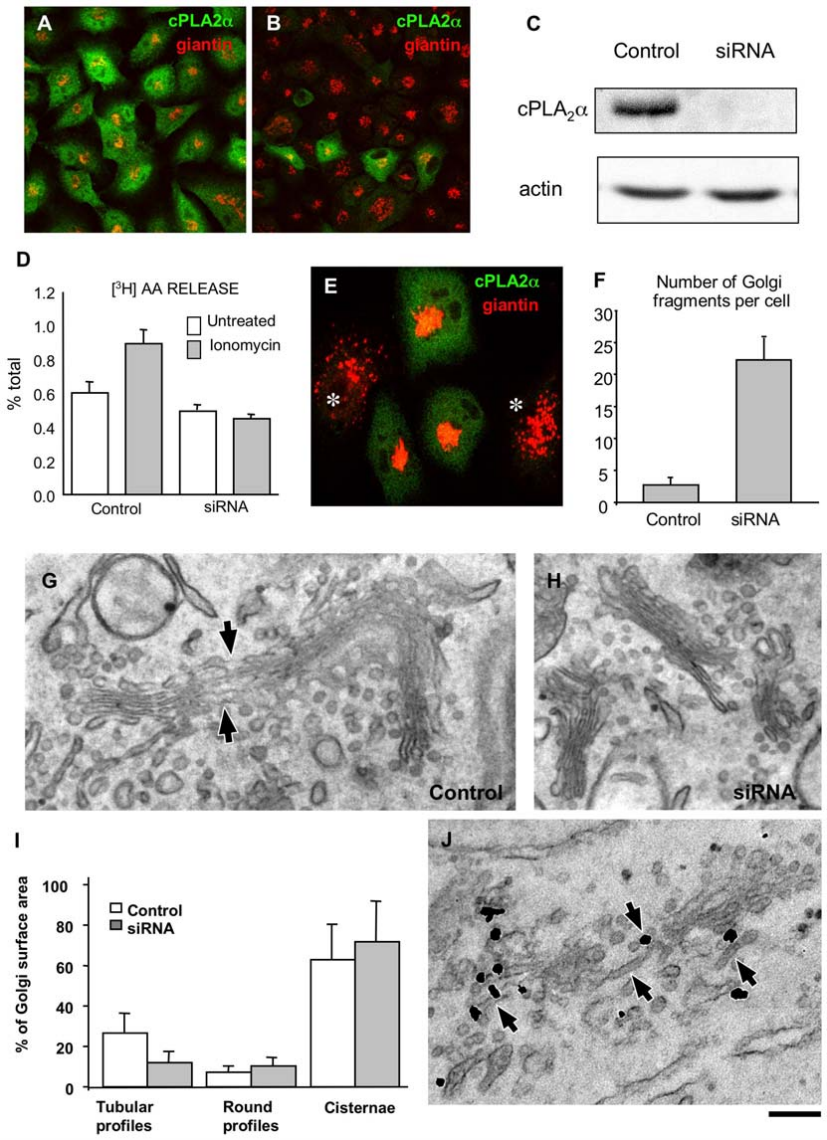
RNAi of cPLA_2_α affects Golgi-associated tubular structures. (A, B) HeLa cells were fixed 72 h after transfection of control scrambled (A) and cPLA_2_α-specific (B) siRNAs and stained for cPLA_2_α and giantin. Confocal microscopy shows a strong reduction in cPLA_2_α labelling in cells treated with the specific siRNAs (B). (C) HeLa cells treated with siRNAs as in (A) and (B) and prepared for western blotting with antibodies against cPLA_2_α and actin. Expression of cPLA_2_α was strongly inhibited, while actin levels remained unaffected. (D) Quantification of cPLA_2_ activity (mean±SD; measured using [^3^H]-AA release; see Materials and Methods) revealed its reduction in cPLA_2_α-siRNAs-treated HeLa cells. (E, F) Control and cPLA_2_-specific siRNAs-transfected HeLa cells. Labelling with an anti-giantin ab (E) and morphometric analysis (F) show extensive fragmentation of the Golgi complex in cells with low cPLA_2_α expression (E, asterisks). (G–I) Control (G, I) and cPLA_2_α-siRNAs-treated HeLa cells (H, I) were fixed and prepared for EM analysis. Tubular structures were seen to connect the Golgi stacks to each other (G, arrows) in control cells but were lost upon cPLA_2_α knock-down (H). Morphometric analysis indicates a reduction in surface area (mean±SD; *n* = 30 stacks) associated with tubular structures in the Golgi complex in cPLA_2_α-siRNAs-treated cells (I). (J) HeLa cells transfected with cPLA_2_α-GFP, fixed and processed for immono-gold EM with an anti-GFP ab. Cells overexpressing cPLA_2_α-GFP show numerous long tubular profiles in the Golgi region (arrows) and partial loss of stack organization. Scale bar, 45 µm (A, B), 22 µm (E), 300 nm (G, H, J).

**Figure 3:**
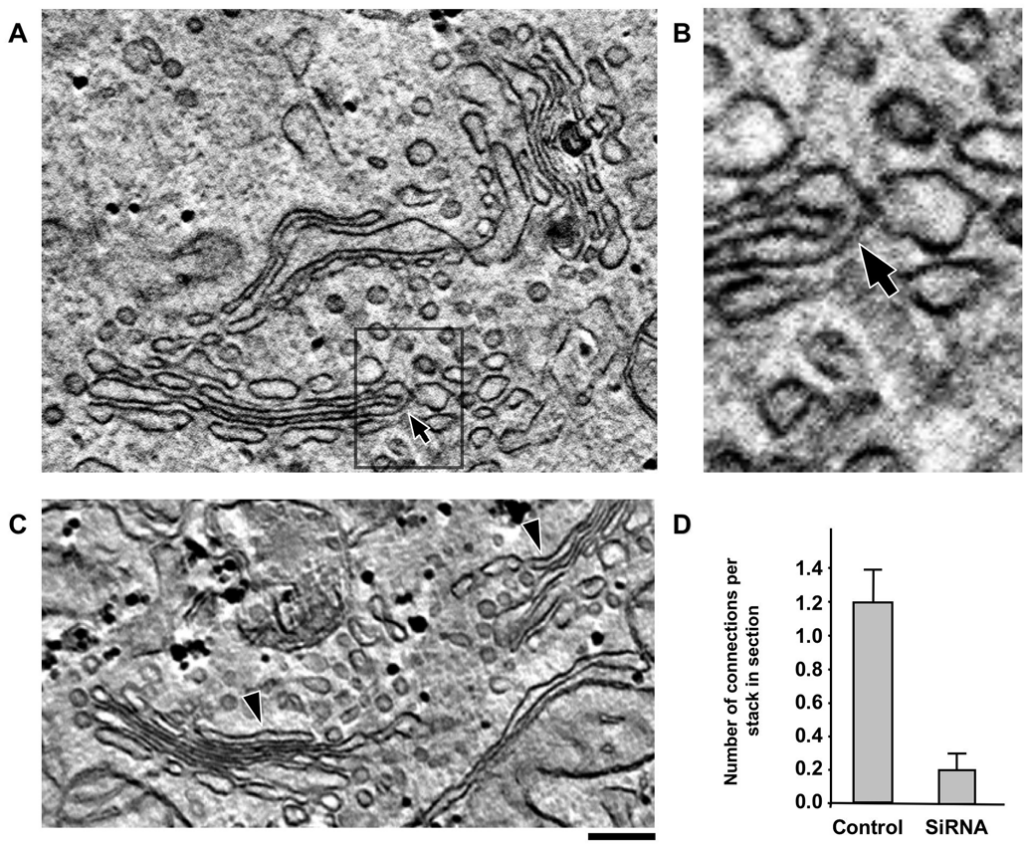
RNAi of cPLA_2_α affects intercisternal connections within the Golgi stack. Control (A, B) and cPLA_2_α-silenced (C) HeLa cells were prepared for EM tomography after chemical fixation. (A, B) The digital slice (A) and zoom image (B; corresponding to the area outlined by black box in A) extracted from the tomogram (see Video S2) shows an intercisternal connection (arrow) bridging cisternae located at the different levels of the stack in control cells. (C) Digital image extracted from the tomogram reveals no bridges between cisternae within the same Golgi stack (see Video S3) as well as a lack of connection between neighbouring stacks (indicated by arrowheads). (D) Quantification of vertical connections per stack in sections (mean±s.e.; *n* = 10 stacks) in EM tomograms (see Materials and Methods), in control and cPLA_2_α-siRNAs-treated HeLa cells. Scale bar, 150 nm (A, C), 50 nm (B).

We next examined whether cPLA_2_α is involved in the formation of intercisternal tubules. Tubules can interconnect the stacked Golgi cisternae in at least two ways: tubular non-compact zones can join adjacent stacks “longitudinally,” to form the continuous Golgi ribbon [1], and tubules can link the Golgi cisternae in the *cis*-*trans* (“vertical”) direction within the same stack, as shown by EM tomography and stereoscopy [12],[18],[21]. To examine the role of cPLA_2_α here, we sought to inhibit/deplete cPLA_2_α by a variety of approaches, while monitoring the presence/formation of Golgi tubules. HeLa cells were first exposed to siRNAs directed against cPLA_2_α. This resulted in a decrease in cPLA_2_α levels, as evaluated by immunofluorescence (Figure 2A and 2B), western blotting (Figure 2C), and cPLA_2_α activity assays under basal and elevated Ca^2+^ conditions (Figure 2D). In these cells, growth was partially inhibited (50%–70%) in the last 24 h of exposure to the siRNAs; however, cell viability did not appear to be affected. In these cPLA_2_α-silenced cells, the Golgi ribbon was disassembled into numerous fragments that remained perinuclear (Figure 2E, asterisks, 2F), as has been previously described upon application of PLA_2_ inhibitors [28],[29]. EM showed that this was due to a suppression of the longitudinal tubular elements (Figure 2G–2I) of the non-compact zones [1], which resulted in the breakdown of the Golgi ribbon into separate stacks (Figure 2H). We then investigated whether this cPLA_2_α deficit also affects vertical intercisternal connections, which are presumably more relevant to *cis*-*trans* transport, using EM tomography (which is required to fully reconstruct these tubular structures [18],[21]). This showed that tubules connecting different cisternae were present within individual Golgi stacks in these cells (Figure 3A and 3B, arrows; Video S2; see also below), as has been previously reported for other cell types [18], and that these tubules were almost completely suppressed by RNA interference (RNAi) of cPLA_2_α (Figure 3C and 3D; Video S3). Other tools that specifically inhibit cPLA_2_α had similar effects: both microinjection of an ab against the catalytic domain of cPLA_2_α (see below) and treatment with the selective inhibitors of cPLA_2_α catalytic activity pyrrophenone and pyrrolidine (not shown) [45],[46] induced a significant fragmentation of the Golgi ribbon corresponding to a reduction in the tubular structures at the EM level (not shown). Of note, the tubules in the non-compact zones and the vertical intercisternal continuities always responded in the same way to a cPLA_2_α deficit, suggesting that they both depend on the activity of cPLA_2_α. Instead, other intracellular tubular structures (such as those of endosomal origin, for example) were not affected by cPLA_2_α depletion (not shown).

We also examined the effects of enhancing the levels of cPLA_2_α by its overexpression. Remarkably, this treatment caused an overall increase in the Golgi tubular elements that was sometimes accompanied by a partial loss of the stack structure (Figure 2J), further supporting the concept that PLA_2_α promotes Golgi tubulation.

A further point was whether the tubulating effects of cPLA_2_α arise from the formation of lysolipids, which can create positive membrane curvature directly [24],[27], or whether they are mediated by the formation of arachidonic-acid (AA) metabolites, perhaps via their signalling function. To test the latter possibility, we used chemical inhibitors to block the main metabolic enzymes of AA [47], the cyclooxygenases (using 50 µm indomethacin and 5 µm ibuprofen for 30 min) and the lipoxygenases (using 10 µm ketokenazol for 30 min). These agents had no influence on Golgi tubule formation (unpublished data), consistent with previously reported observations [28]. Moreover, the addition of AA to cPLA_2_α-siRNAs-treated cells did not counteract the “anti-tubular” effects of the cPLA_2_α deficit. Altogether, these data suggest that cPLA_2_α is required to support tubulation and that its effects are mediated via the formation of lysolipids [24],[25],[27],[28].

**Figure 4:**
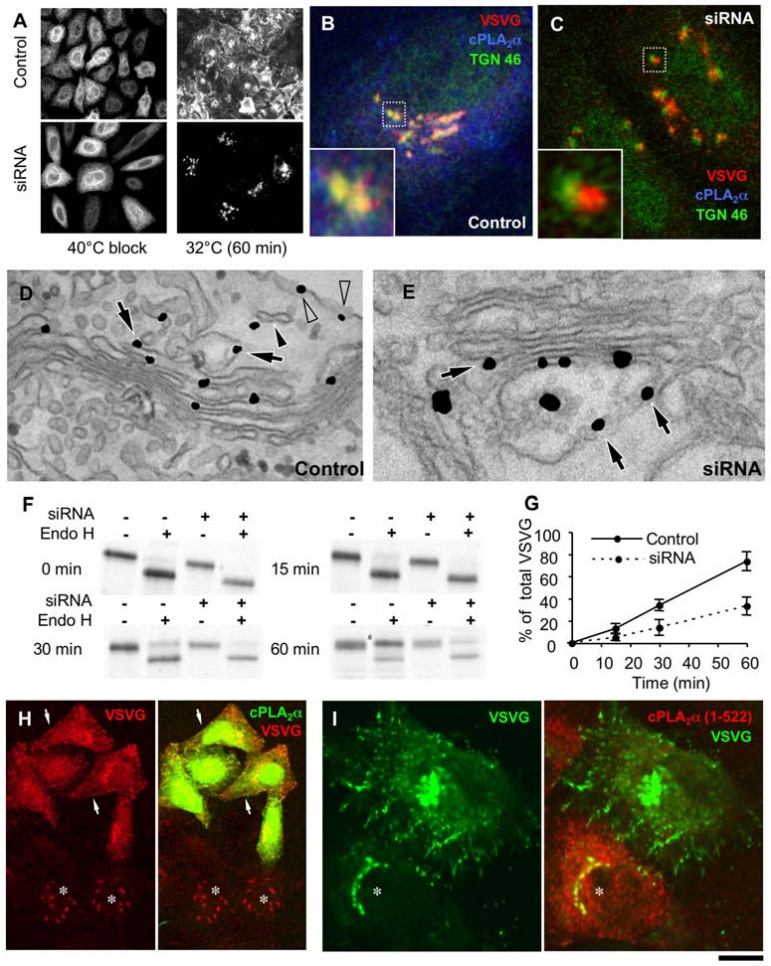
RNAi of cPLA_2_α inhibits intra-Golgi transport of VSVG. (A–E) Control (A, B, D) and cPLA_2_α-siRNAs-treated HeLa cells (A, C, E) were infected with VSV and kept at 40°C for 3 h to accumulate VSVG within the ER. The cells were fixed directly at the end of the block (A) or after a 60-min (A) or 45-min (B–E) temperature shift to 32°C. (A) The cells were labelled with an anti-VSVG ab, showing that after accumulation within the ER, VSVG was efficiently exported to the plasma membrane in control cells and blocked in the Golgi complex in cPLA_2_α-siRNAs-treated cells. (B–E) The cells were triple labelled with anti-VSVG, anti-cPLA_2_α, and anti-TGN46 antibodies (B, C) or prepared for immuno-EM using the nanogold protocol (D, E). In control cells, VSVG showed good colocalization with TGN46 in the Golgi area (B, inset); this colocalization was poor in cPLA_2_α-siRNAs-treated cells (C, inset). In control cells, EM showed little VSVG in the *cis* portion of the stack (7.8%), with most (61%) within *trans*-Golgi compartments (D, arrows), post-Golgi carriers (D, filled arrowhead), and at the plasma membrane (D, empty arrowhead). In cPLA_2_α-siRNAs-treated cells, most of the VSVG (62%) remained within the swollen *cis* portion of the stack (E, arrows). (F, G) Control and cPLA_2_α-siRNAs-treated HeLa cells infected with VSV were metabolically labelled with [^35^S]-methionine and chased at 32°C. At the indicated times, the cells were solubilized and digested with endoglycosidase H (Endo-H), which cleaves sugar chains built on the proteins early in the secretory pathway (i.e., before their processing by the medial Golgi enzyme mannosidase-II, which convert sugars into an Endo-H resistant form). The cell lysates were then separated by SDS-PAGE, and the gels scanned (F). The percentages of the Endo-H-resistant form of VSVG with respect to the total amounts of VSVG were quantified (G) using a FUJIFILM imager. The data indicate that VSVG processing to its Endo-H resistant form (which occurs in the medial Golgi) was reduced when cPLA_2_α was silenced. (H) cPLA_2_α-siRNAs-treated cells were infected with VSV, microinjected with recombinant cPLA_2_α during the 40°C block, and fixed 45 min after the block release at 32°C. The cells were then stained with anti-VSVG and anti-cPLA_2_α antibodies and observed under the confocal microscope. VSVG was delivered to the plasma membrane after cPLA_2_α microinjection (arrows) but remained in the Golgi in noninjected cells (asterisks). (I) HeLa cells were transfected with VSVG-GFP and the dominant-negative cPLA_2_α(1–522) isoform, subjected to a 40°C block, and fixed 45 min after the temperature shift to 32°C. Confocal images reveal VSVG-GFP blocked in the Golgi complex in cPLA_2_α(1–522)-expressing cells (asterisks). Scale bar, 60 µm (A), 6 µm (B, C), 270 nm (D), 200 nm (E), 15 µm (H), 8.5 µm (I).

**Figure 5:**
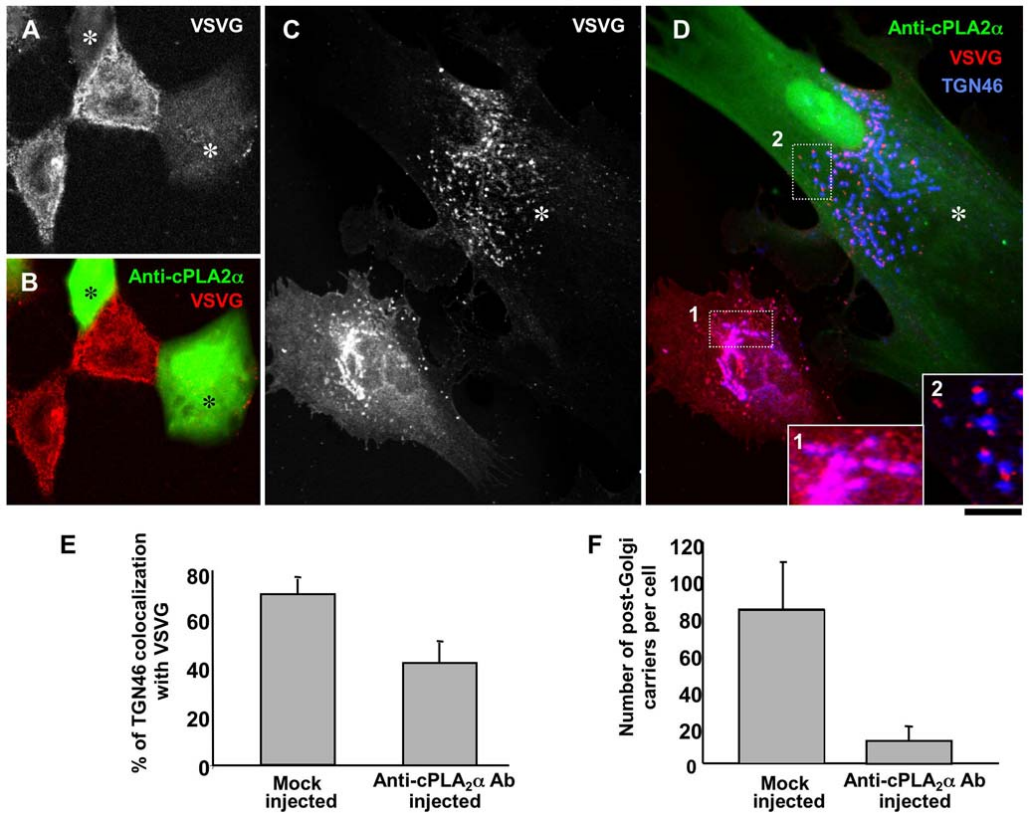
Microinjection of an anti-cPLA_2_α ab inhibits intra-Golgi transport. (A–F) HFs were infected with VSV and kept at 40°C for 3 h to accumulate VSVG in the ER. The cells were microinjected with an anti-cPLA_2_α ab (mixed with FITC dextran) during the block, then shifted to the permissive temperature (32°C) and fixed at different times. (A, B) Staining of non-permeabilized cells with an ab against the ectodomain of VSVG 60 min after the 40°C block release shows reduced VSVG at the cell surface in microinjected cells (asterisks). (C, D) Microinjection with an anti-cPLA_2_α ab (asterisks) induces fragmentation of the Golgi complex, prevents delivery of VSVG to the plasma membrane, and blocks VSVG within the early Golgi compartment without showing overlap with TGN46 (compare insets 1 and 2 in panel D, which correspond to dashed boxes in the control and injected cells). (E, F) Morphometric quantification shows poor colocalization of TGN46 with VSVG (E; mean±SD; *n* = 50 cells) and a reduction in the number of post-Golgi carriers (F; mean±SD; *n* = 50 cells) in anti-cPLA_2_α-ab-microinjected cells, compared to mock-injected cells. Scale bar, 30 µm (A, B), 7.5 µm (C, D).

We thus turned to investigate whether disassembly of intercisternal connections affects transport of cargo proteins across the Golgi complex. To test this, we first suppressed intercisternal connections by silencing cPLA_2_α, and then monitored the effects of this treatment on intra-Golgi transport using the VSVG-synchronized transport assay. We thus infected cPLA_2_α-depleted cells with VSV and accumulated VSVG in the ER at 40°C, and then we released the traffic block at 32°C. VSVG reached the Golgi complex apparently normally, but then it accumulated at the *cis*-Golgi pole (also inducing a moderate swelling of the *cis* cisternae) and did not proceed through the Golgi complex (Figure 4A–4E). Compatible results were obtained using biochemical transport assays (see Materials and Methods) (Figure 4F and 4G). Rescuing the cPLA_2_α activity in cPLA_2_α-siRNAs-treated cells by microinjection of recombinant cPLA_2_α resulted in the reactivation of VSVG trafficking (Figure 4H). Further along this line, an arrest of VSVG in the Golgi complex was seen also when the cells were transfected with a dominant-negative cPLA_2_α mutant [48] (Figure 4I), and when cPLA_2_α was acutely inhibited by the microinjection of antibodies against the catalytic portion of cPLA_2_α (Figure 5) or by specific inhibitors (not shown). Notably, these inhibitory effects were marked but not complete (up to 60%–80%; see Figure 4), possibly explaining the lack of visible toxicity (at least within our experimental time frame).

A possible concern here is that these experiments were carried out using a synchronization protocol that involves the sudden arrival of a large cargo load at the Golgi complex, raising the possibility that cPLA_2_α-dependent connections might have a role only under conditions of Golgi overload. We therefore examined whether the inhibition of cPLA_2_α would cause similar effects during more “physiological” non-synchronized trafficking. To this end, we infected cPLA_2_α-silenced cells with VSV and kept them at 32°C, to allow VSVG to continuously exit the ER. As was seen during the 40–32°C synchronized pulse, VSVG reached the Golgi complex but did not traverse it (see below), indicating that the role of cPLA_2_α in intra-Golgi trafficking is not limited to conditions of cargo overload.

The transport of other cargo proteins was also examined using cPLA_2_α silencing. Ablation of cPLA_2_α in HFs resulted in a strong delay of PC-I transport at the level of the Golgi complex (unpublished data). The use of cyclooxygenase and lipooxygenase inhibitors had no effects on trafficking, and AA addition did not reverse the transport block in cPLA_2_α-siRNAs-treated cells, again in parallel with the above effects on Golgi tubules. Thus, the induction of a cPLA_2_α deficit and the attendant disassembly of intercisternal connections appear to be associated with the inhibition of intra-Golgi trafficking of different classes of cargo proteins.

**Figure 6:**
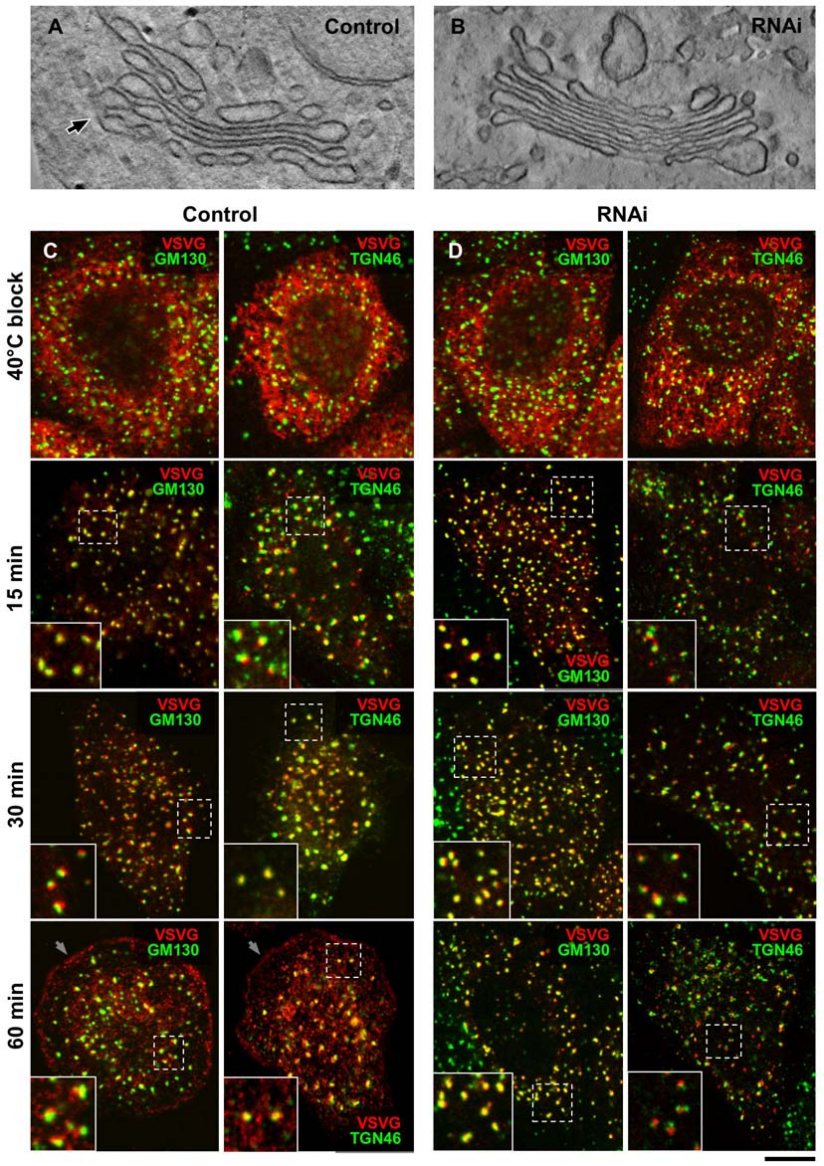
RNAi of cPLA_2_α affects intercisternal connections and transport within NZ-induced Golgi ministacks. (A, B) Control (A) and cPLA_2_α-silenced (B) HeLa cells were treated with 30 µM NZ for 3 h, then fixed and prepared for EM tomography. The digital slices extracted from the tomograms (see Videos S4 and S5) show an intercisternal connection (arrow) bridging cisternae located at the different levels of the stack in control cells (A) and reveals no bridges between cisternae within the Golgi ministack in silenced cells (B). (C, D) Control (C) and cPLA_2_α-siRNAs-treated HeLa cells (D) were infected with VSV and kept at 40°C for 3 h in the presence of NZ to disassemble Golgi into ministacks and accumulate VSVG within the ER. The cells were fixed directly at the end of the block or at indicated times after the temperature shift to 32°C. The cells were double labelled with an anti-VSVG and either GM130 (*cis*-Golgi marker) or TGN46 (*trans*-Golgi marker) and investigated under confocal microscopy. In control cells (C), after release of 40°C block, VSVG moved from the ER to the Golgi ministacks where it first colocalized with GM130 and then with TGN46 (see insets) and finally arrived to the cell surface (arrows). In contrast, cPLA_2_α-silenced cells show VSVG within the GM130-positive compartment of ministacks (see insets) even 60 min after release from the ER, with little or no VSVG detected both in the TGN46-positive compartment and at the plasma membrane. Scale bar, 150 nm (A, B), 6 µm (C, D).

A further question is which type of connection is required for trafficking. Our data indicate that the inhibition of cPLA_2_α disrupts the “vertical” (*cis*-*trans*) and “longitudinal” (inter-stack) tubular connections equally (see Figures 2 and 3). Thus, although it may appear logical to assume that the vertical connections are those relevant for transport, a role for the longitudinal connections cannot be formally excluded by the above data. To resolve this issue, we used a Golgi system that contains vertical, but lacks horizontal, connections, and tested whether transport through such a system is sensitive to the ablation of cPLA_2_α. A Golgi complex with only vertical connections can be generated by nocodazol (NZ) treatment, which results in the fragmentation of the Golgi ribbon into isolated (non-horizontally connected) stacks [18]. Any transport-relevant role of longitudinal connections can be excluded in this system. Control and cPLA_2_α-deficient cells were subjected to 3 h of 30 µM NZ treatment and processed for EM tomography. As expected, vertical connections were revealed in stacks of control cells (Figure 6A; Video S4), while in silenced cells, these connections were almost absent (Figure 6B; Video S5). To test the efficiency of transport under these conditions, the cells were infected with VSVG and exposed to NZ during the 40°C block, and then shifted to 32°C to activate transport. Figure 6C shows that in control cells, VSVG moved to the plasma membrane first through GM130-positive and then through TGN46-positive compartments of the Golgi stacks, as has been previously reported [18]. In contrast, in cPLA_2_α-silenced cells, VSVG was retained in the Golgi stacks, where it showed strong overlap with the *cis*-Golgi marker (GM130) even 60 min after release from the ER (Figure 6D). Thus, the disruption of vertical intercisternal bridges by cPLA_2_α silencing inhibited the progression of cargo across NZ-induced isolated stacks, which are devoid of horizontal connections. This provides evidence that it is the intercisternal connections of the vertical type that are required for intra-Golgi transport (see Discussion).

### The Catalytic Activity of cPLA_2_α Is Required to Support Intra-Golgi Transport

Our experiments with specific cPLA_2_ chemical inhibitors (see above) taken together with already published observations [27] suggest that the changes in lipid geometry during Golgi tubulation require PLA_2_ catalytic activity. Nevertheless, given that the C2 domain of cPLA_2_α inserts deep into the membrane bilayer [49] and could therefore be classified among the membrane-bending protein modules [50], we wanted to determine whether it is indeed the catalytic activity of cPLA_2_α, rather than the insertion of this enzyme into the Golgi membranes, that is responsible for the generation of tubules and transport across the Golgi.

**Figure 7:**
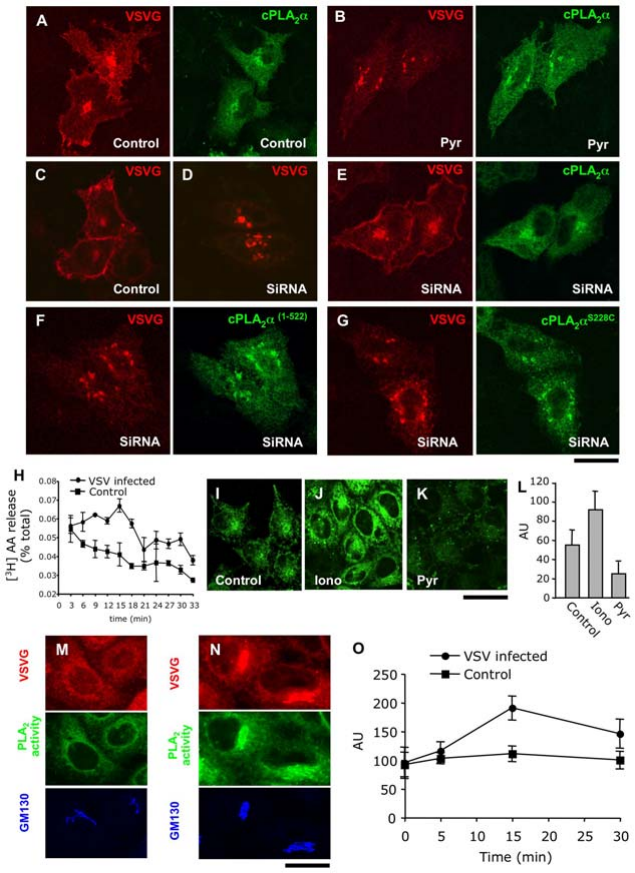
The catalytic activity of cPLA_2_α is required to support intra-Golgi transport. (A, B) HeLa cells were co-transfected with cPLA_2_α-GFP and VSVG-Cherry and exposed to the 40°C block to accumulate VSVG-Cherry within the ER. Pyrrolidine (1 µM; Pyr) was added to the cells (B) 15 min before block release. Then cells were shifted to 32°C in the absence (A) or in the presence (B) of pyrrolidine for 45 min, fixed, and investigated under confocal microscopy. In control cells, VSVG-Cherry was detected at the cell surface (A) and its delivery to the PM was inhibited in pyrrolidine-treated cells (B) ,while cPLA_2_α-GFP recruitment to the Golgi membranes was not affected by inhibitor treatment (compare A and B). (C–G) Control (C) and cPLA_2_α-silenced (D–G) mouse MC3T3 cells were transfected with VSVG-Cherry alone (C, D) or in combination with the following human cPLA_2_α constructs: cPLA_2_α-GFP (E), cPLA_2_α^(1–522)^ (F), cPLA_2_α^S228C^ (G). The cells were subjected to 40°C block, then shifted to 32°C (to activate VSVG-Cherry exit from the ER) for 45 min, fixed, and stained with an anti-cPLA_2_α ab. Confocal microscopy revealed efficient delivery of VSVG-Cherry to the PM in control cells (C), while in silenced cells, most of the VSVG-Cherry remained within the fragmented Golgi complex (D). Transfection of wild-type cPLA_2_α-GFP rescued VSVG-Cherry delivery to the cell surface in cPLA_2_α-silenced cells, while expression of the catalytically inactive mutants cPLA_2_α^(1–522)^ (F) or cPLA_2_α^S228C^ (G) did not reactivate VSVG-Cherry transport. (H) PCCL3 cells were loaded with [^3^H]-AA (see Materials and Methods). Part of the loaded cells was infected with VSV. Then infected and noninfected (control) cells were exposed to the 40°C block, washed, and shifted to 32°C. Fresh medium was added to the cells for 3 min time intervals and then collected. The [^3^H]-AA released into the medium over each 3 min-long interval was measured (see Materials and Methods). Quantification of the AA release (mean±SD; *n* = 6 experiments) revealed increase of PLA_2_ activity (with the peak at 15 min) in VSV infected cells as compared to control. (I–L) HeLa cells were loaded with bis-BODIPY FL C_11_-PC (see Materials and Methods) and fixed directly (I) or 15 min after incubation with either 5 µM ionomycin (J) or 1 µM pyrrolidine (K). The fluorescent signal indicating PLA_2_ activity was detected by confocal microscopy both in the perinuclear region and the periphery of control cells (I). This signal increased after ionomycin treatment (J), but decreased after pyrrolidine addition (K), as also revealed by quantification (L) of the mean fluorescence per cell (mean±SD; *n* = 20 cells). (M, O) HeLa cells were infected with VSV, kept at 40°C for 3 h, and loaded with bis-BODIPY FL C_11_-PC at the end of 40°C block. The cells were then fixed directly at the end of the 40°C incubation (M) or at different time intervals after temperature shift to 32°C (N, O). The cells were then stained with antibodies against VSVG and GM130 (Golgi marker). Confocal microscopy revealed only moderate PLA_2_ activity overlapping with the Golgi when VSVG resided within the ER (M) while activation of VSVG transport through the Golgi (15 min after release from the ER) induced an increase in the BODIPY signal in the Golgi area (N). Quantification of the mean BODIPY fluorescence per cell (mean±SD; *n* = 20 cells) indicates an increase in PLA_2_ activity in cells actively transporting VSVG, as compared to uninfected (control) cells (O). Scale bar, 8.5 µm (A–G), 16 µm (I–K), 7 µm (M, N).

We first tested whether cPLA_2_α maintains its ability to bind to the Golgi complex in the presence of chemical inhibitors that suppress intra-Golgi transport. VSV-infected HeLa cells were treated with pyrrolidine, and the localization of cPLA_2_α was monitored during a VSVG traffic pulse. cPLA_2_α translocated to the Golgi complex to the same extent in control and inhibitor-treated cells, but only the latter showed VSVG retention at the Golgi (Figure 7A and 7B). Second, we examined the effects of two cPLA_2_α mutants, cPLA_2_α^(1–522)^ and cPLA_2_α^S228C^, which lack PLA_2_ catalytic activity and yet show normal binding to membranes [48],[51]. cPLA_2_α^(1–522)^ is a deletion mutant that lacks an amino acid (Asp549) essential for enzyme activity [52]. Of note, cPLA_2_α^(1–522)^ can be produced endogenously by caspase-mediated cleavage at Asp^522^ during apoptosis [48], and it can act as a dominant-negative mutant of cPLA_2_α [48]. The cPLA_2_α^S228C^ mutant contains a single point mutation in the active site, again resulting in a complete loss of cPLA_2_α enzymatic activity [51].

Each mutant was transfected into cPLA_2_α-silenced cells and compared to wild-type cPLA_2_α for its ability to rescue the transport block induced by cPLA_2_α ablation and to translocate to the Golgi complex. For the transport experiments, mouse cells were transfected with VSVG carrying a red fluorescent tag (VSVG-Cherry) and subjected to the transport-synchronization protocol. VSVG-Cherry was efficiently delivered to the surface in control cells (Figure 7C) but not in cPLA_2_α-silenced cells, as expected (Figure 7D). The cPLA_2_α-silenced cells were then transfected with either the mutants or the wild-type cPLA_2_α. While the latter efficiently rescued VSVG-Cherry transport to the cell surface (Figure 7E), as expected, neither cPLA_2_α^(1–522)^ nor cPLA_2_α^S228C^ modified the transport block (Figure 7F and 7G). However, both cPLA_2_α mutants translocated to the Golgi complex as efficiently as wild-type cPLA_2_α (Figure 7F and 7G). Therefore, these collective results indicate that the catalytic activity of cPLA_2_α, rather than the ability of this enzyme to translocate to Golgi membranes, is required to support transport across the Golgi complex.

Finally, we sought to directly monitor the increase in cPLA_2_α activation that based on the above data should occur during cargo trafficking through the Golgi complex. First, we used a classical PLA_2_ activity assay based on the release of [^3^H]-AA from AA-prelabelled cells [53]. A potential problem here is that during cargo trafficking, only a fraction of the total cellular cPLA_2_α is bound to the Golgi complex (which represents, in turn, less than 5% of the cellular membranes). Thus, the increase in AA release over basal values might be very small. To overcome these problems, we used two approaches.

For the first, in addition to HeLa cells, we used a cell line that has been previously characterized in our laboratory to be an efficient AA releaser (PCCL3 cells) [46]. Both cell types were loaded with [^3^H]-AA, infected with VSV, and subjected to a 40–32°C transport synchronization protocol. When they were shifted from 40°C to the permissive temperature of 32°C, the VSVG expressing PCCL3 cells showed a modest but statistically significant increase in AA release over that seen in control cells (Figure 7H). This increase coincided in time with VSVG transit through the Golgi complex. HeLa cells showed a trend in the same direction, which, however, was not statistically significant. To overcome this difficulty with HeLa cells, we used here a second approach based on the fluorogenic phosphatidylcholine analogue (bis-BODIPY FL C_11_-PC) as a sensor of local changes in PLA_2_ activity [54]. The hydrolysis of this lipid by PLA_2_ enzymes results in generation of fluorescent products by fluorescence dequenching [54]. After loading the bis-BODIPY FL C_11_-PC, HeLa cells at steady-state showed a diffuse (ER-like) fluorescent signal in the cell periphery (indicating ongoing PLA_2_ activity), and a clearer signal in the perinuclear (Golgi) area (as assessed by confocal microscopy, Figure 7I). In control experiments, the application of the calcium ionophore ionomycin, which strongly stimulates cPLA_2_α [31], markedly increased this signal both at the cell periphery and in the perinuclear region, while the cPLA_2_α inhibitor pyrrolidine reduced overall BODIPY fluorescence (Figure 7J–7L), indicating that the probe functions as expected under our conditions. Then, the cells were exposed to the 40–32°C VSVG synchronized traffic pulse. During the 40°C block (Figure 7M) the cells did not show any significant concentrating of fluorescence signal in the Golgi area (consistent with the lack of transport through the Golgi and of cPLA_2_α recruitment). When the traffic block was released, the fluorescence signal increased selectively in the Golgi area, to nearly 2-fold the control (Figure 7N and 7O).

Thus, taken together, the AA release and the microscopy data suggest that the catalytic activity of cPLA_2_α increases during the passage of cargo through the Golgi complex and that this activity is required for transport across the Golgi stack.

### Suppression of cPLA_2_α Activity Does Not Inhibit Golgi Vesicle Formation

A series of control experiments was then carried out. In the first, we asked whether the inhibition of cPLA_2_α activity might have an effect on the Golgi COPI vesicles. We thus inhibited/depleted cells of cPLA_2_α and examined the features of the Golgi vesicles as well as on the dynamics of the COPI machinery in these cells. cPLA_2_α silencing affects neither the number nor the morphology of Golgi vesicles (Figure 2H and 2I). We also blocked vesicle fusion with their target membranes and monitored the kinetics of vesicle accumulation as an indicator of the rate of vesicle formation. This was achieved via inhibition of αSNAP (one of the main membrane fusion factors) by incubating permeabilized cells with an L294A αSNAP mutant that blocks fusion [55],[56]. L294A αSNAP induced an accumulation of Golgi vesicles, as expected. This accumulation was the same in the absence and presence of the cPLA_2_α inhibitor (Figure S1A–S1E). Also, the machinery responsible for COPI vesicle formation was not affected by the cPLA_2_α inhibitor, as judged by the COPI and ARF1 dynamics of association with Golgi membranes in live cells (Figure S1F–S1K). Finally, we looked at the effects of cPLA_2_α silencing on a known COPI-vesicle-dependent trafficking step: the recycling of the KDEL receptor (KDELR) from the Golgi complex to the ER. For this, we used a well-characterized assay based on a KDELR-VSVG chimera [57]. This assay indicated that the KDELR recycles from the Golgi complex to the ER equally well in control and cPLA_2_α-siRNAs-treated cells (see below, Figure S2A–S2D), again indicating that the COPI machinery is not inhibited by a cPLA_2_α deficit.

Thus, treatments that block cPLA_2_α suppress the formation of intercisternal tubules while having no apparent inhibitory effects on Golgi-associated COPI vesicles (which presumably rely mostly on coat proteins for their curvature; [23]).

### Specificity of the Effects of cPLA_2_α Inhibition on Different Trafficking Steps

We also examined the specificity of the effects of cPLA_2_α on several transport steps. Since cPLA_2_α is recruited selectively to the Golgi complex upon activation of transport and its inactivation selectively suppresses Golgi tubule formation, its effects on trafficking should be restricted to the Golgi complex. In contrast, some relatively nonspecific inhibitors of many PLA_2_ isoforms used previously, such as ONO [58], have been reported to block transport at multiple segments of the exocytic and endocytic transport pathways that rely on tubular transport intermediates (reviewed in Brown et al. [27]). To address this apparent discrepancy, we compared the effects of cPLA_2_α RNAi and of pyrrophenone with those reported for ONO [28],[29].

First, we examined the effects of silencing cPLA_2_α on several transport steps. These included retrograde transport of the KDELR from the Golgi complex to the ER (as above; Figure S2A–S2D) plus endocytosis and recycling of transferrin to the plasma membrane (Figure S2E and S2F), and endocytosis of wheat-germ agglutinin lectin uptake and its transport to the *trans*-Golgi network (TGN) (Figure S2G and S2H). None of these steps was affected by cPLA_2_α silencing. We also examined the TGN-to-plasma-membrane transport of VSVG after a 20°C transport block (at this temperature, VSVG is arrested and accumulates both in the TGN and in the medial-*trans* Golgi cisternae [4]). When the 20°C block was released in inhibitor-treated cells, a large fraction of VSVG reached the plasma membrane normally (presumably from the TGN), while the remaining fraction (presumably residing in the medial-*trans* cisternae) remained trapped in the Golgi complex (not shown), consistent with an effect of cPLA_2_α silencing on intra-Golgi trafficking (see above) and with a lack of effect on TGN-to-plasma-membrane transport. Also, transport from the ER to the Golgi was not affected by cPLA_2_α silencing, as shown above (see Figure 4). Furthermore, the labelling of different proteins that reside in the endocytic compartments (Figure S2I–S2L) or at the ER/Golgi interface (Figure S2M–S2P) showed no significant changes after this specific cPLA_2_α silencing. Thus, these data confirm the selectivity of the cPLA_2_α role in intra-Golgi trafficking.

We then compared the above effects with those of ONO, a relatively nonspecific drug that inhibits several PLA_2_ isoforms [27]. ONO inhibited the transport of VSVG (Figure S3A–S3F, S3K, and S3L) and PC-I (Figure S3G–S3J). Moreover, ONO induced the structural changes expected of PLA_2_ inhibition; namely, fragmentation of the Golgi ribbon (Figure S4A and S4B) and suppression of Golgi-associated tubular elements (Figure S4C–S4E), including intercisternal connections (as revealed by EM tomography) (Figure S4F and S4G; Videos S6 and S7). These effects were reversible, as ONO wash-out resulted in a rapid reappearance of bridges connecting cisternae within the stack (Figure S4H–S4K; Video S8), which coincided with reactivation of transport through the Golgi complex (Figure S3M–S3O). These effects mimic those due to cPLA_2_α inhibition; however, in addition to these, ONO had effects that were not seen in cPLA_2_α-silenced cells, including the suppression of tubular structures in transferrin-containing early endosomes (not shown). Moreover, ONO has been shown by others to suppress the recycling of transferrin from the endosomes to the plasma-membrane [59], as well as retrograde transport from the Golgi complex back to the ER [60]. It is possible that as an inhibitor of many PLA_2_ isoforms, ONO has generalized effects on different membrane tubules because these depend on activities of different PLA_2_ enzymes [27], while cPLA_2_α is Golgi specific (see Discussion).

### Other PLA_2_ Enzymes Support Trafficking in Cells Derived from cPLA_2_α Knock-Out (KO) Mice

**Figure 8:**
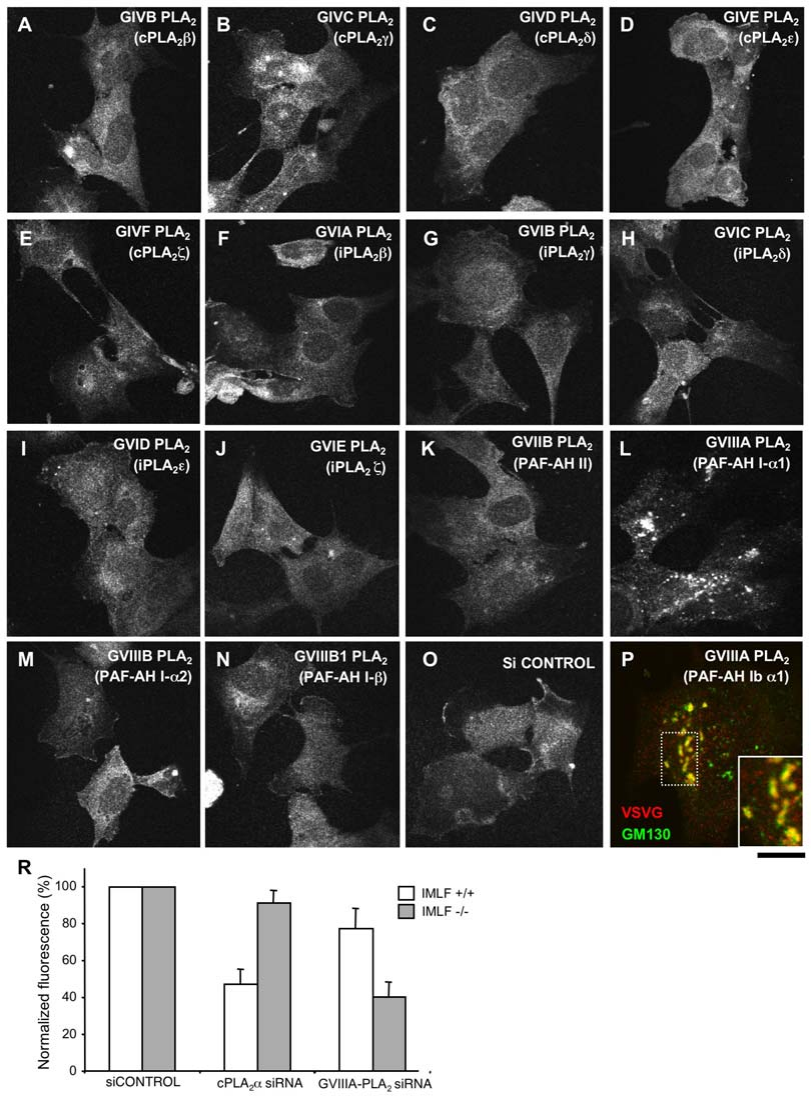
GVIIIA-PLA_2_ inhibits transport of VSVG in immortalized murine lung fibroblasts from cPLA_2_α-KO mice. (A–P) IMLFs from cPLA_2_α KO mice (IMLFs^−/−^) were incubated with siRNAs specific for the different cytosolic PLA_2_ enzymes, and control siRNAs, for 72 h (A–P; as indicated). The cells were then infected with VSV, kept at 40°C for 3 h to accumulate VSVG in the ER, and incubated in fresh medium at 32°C for 60 min before fixing. The fixed cells were either stained with only an anti-VSVG ab (A–O) or double labelled for VSVG and GM130 (P). VSVG was efficiently exported to the plasma membrane in cells incubated with either control (O) or most of the PLA_2_-isoform-specific siRNAs (A–K, M–O). However, RNAi of GVIIIA-PLA_2_ induced accumulation of VSVG within the Golgi complex of IMLFs (L). Notably, VSVG always overlapped strongly with GM130 (see inset in P) in cells incubated with GVIIIA-PLA_2_-specific siRNAs, suggesting that VSVG is trapped within the *cis*-Golgi compartment. (R) IMLF^−/−^ and control lung fibroblasts from mice expressing cPLA_2_α (IMLFs^+/+^) were incubated with siRNAs specific for either cPLA_2_α or GVIIIA-PLA_2_ and control siRNAs for 72 h. The cells were then infected with VSV, subjected to the 40°C block with its further release for 60 min and fixed. VSVG was detected at the surface of IMLFs^−/−^ and IMLFs^+/+^ using an ab against its ectodomain. Afterwards, the cells were permeabilized and incubated again with an anti-VSVG ab to reveal the total pool of VSVG within the cell. Then fluorescense intensities of surface and total VSVG were evaluated, expressed as a ratio, and normalized to the control. This quantification reveals that cPLA_2_α silencing significantly inhibits VSVG transport in IMLFs^+/+^ (but not in IMLFs^−/−^), while GVIIIA-PLA2 RNAi strongly affects VSVG transport in IMLFs^−/−^ and only slightly in IMLFs^+/+^. Scale bar, 18 µm (A–O), 7.2 µm (P).

An apparent difficulty in this study is that cPLA_2_α KO mice show reduced fertility but, surprisingly, no other major phenotypes [61],[62]. We examined whether cells obtained from these mice show transport abnormalities. To test this, immortalized lung fibroblasts (IMLFs) from cPLA_2_α KO mice (IMLFs^−/−^) or control mice (IMLFs^+/+^) were infected with VSV and subjected to the 40–32°C transport synchronization protocol (Figure S5). No significant differences in VSVG transport were detected between IMLFs^+/+^ and IMLFs^−/−^ (Figure S5A, S5C, S5E, S5G), except that while intra-Golgi transport in IMLFs from control mice showed the expected inhibition in the presence of the specific cPLA_2_α inhibitor pyrrophenone, the IMLFs from KO mice were insensitive (Figure S5B, S5D, S5F, and S5H). This suggests that the latter cells had developed an adaptive mechanism to compensate for the loss of cPLA_2_α (and, incidentally, confirms the specificity of pyrrophenone). We considered the possibility that this mechanism might be based on other PLA_2_s. To verify this, we used siRNAs to screen for the roles of all of the cytosolic PLA_2_ proteins from Groups IV, VI, VII, and VIII of the PLA_2_ superfamily [30] in VSVG transport in these cPLA_2_α KO cells (Figure 8). The efficiency of siRNA delivery was checked with fluorescent siGLO. Among these siRNAs, only those silencing the Ca^2+^-independent Group VIIIA (GVIIIA)-PLA_2_ inhibited VSVG transport, resulting in accumulation of cargo within the Golgi complex (Figure 8L and 8P). Strikingly, this PLA_2_ isoform has been detected by others at the Golgi membranes and appears to be important for maintenance of the tubular elements of the Golgi complex (W. Brown, personal communication). We also characterized the effects of the GVIIIA-PLA_2_ ablation in IMLFs^−/−^ and saw clear similarities with the effects of cPLA_2_α silencing in “normal” cells. The Golgi remained perinuclear, but the ribbon underwent fragmentation (i.e., it exhibited numerous breaks; Figure 8P), presumably due to the loss of the tubular elements connecting cisternae. VSVG reached the Golgi complex normally, but to a large extent remained trapped in the Golgi complex, where it showed substantial overlap with the *cis*-Golgi marker GM130 (Figure 8P) (i.e., it remained in the *cis*-Golgi) and showed a marked delay of protein progression across the stack. It is thus likely that GVIIIA-PLA_2_ is responsible for compensating for the cPLA_2_α deficit in KO cells (or animals) and for supporting transport through the Golgi complex in these cells.

**Figure 9:**
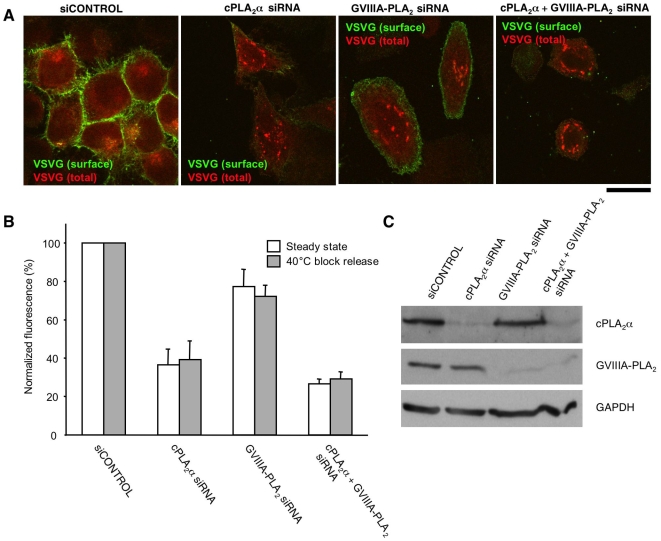
VSVG transport efficiency in HeLa cells after depletion of cPLA_2_α and/or GVIIIA-PLA_2_. HeLa cells were incubated with control siRNAs or siRNAs specific for cPLA_2_α, GVIIIA-PLA_2_, or both for 72 h. (A) Cells exposed to these siRNAs were infected with VSV and kept for 6 h at 32°C to allow continuous synthesis and transport of VSVG through the secretory pathway (“steady-state” conditions). Then the cells were fixed and VSVG at the cell surface was stained with an ab against its ectodomain. Afterwards, the cells were permeabilized and incubated again with an anti-VSVG ab to reveal the total pool of VSVG within the cell. Scale bar, 10 µm. (B) Cells were treated as in A or subjected to the 40°C block and then shifted to the permissive temperature of 32°C for 60 min (“traffic wave” conditions), fixed, and labelled for surface and total VSVG as described above. Then the fluorescence intensities of surface and total VSVG were evaluated, expressed as a ratio, and normalized to the control. This quantification reveals that under both “steady-state” and “traffic-wave” conditions, cPLA_2_α silencing inhibits VSVG transport much more strongly than GVIIIA-PLA_2_ depletion (see also images in panel A). RNAi of both PLA_2_ enzymes, in turn, shows a slightly stronger inhibitory effect on VSVG transport over cPLA_2_α silencing alone. (C) Cells incubated with different siRNAs (as described above) were subjected to western blotting with antibodies against either cPLA_2_α or GVIIIA-PLA_2_ or GAPDH, as indicated.

We also asked whether and to what extend GVIIIA-PLA_2_ is involved in the regulation of transport in other cell lines with normal levels of cPLA_2_α by evaluating transport both in control IMLFs^+/+^ and in HeLa cells depleted in GVIIIA-PLA_2_. The silencing of GVIIIA-PLA_2_ inhibited VSVG transport less markedly than that of cPLA_2_α, and the double knock-down of these enzymes was slightly more effective than that of cPLA_2_α depletion alone (Figure 9), indicating that GVIIIA-PLA_2_ has a subsidiary role in normal cells.

Notably, these experiments were carried out under conditions of both synchronized (high load) and non-synchronized (low load) trafficking with similar results. Also notably, the traffic inhibition was marked (up to 80%), but not complete, even in double KD cells. This could be due either to incomplete silencing or to some further compensatory effects, or also to the presence of redundant transport mechanisms. As a consequence, silenced cells can survive, although their rate of growth was significantly decreased (by 60%–80%) in the last 24 h with the siRNAs, probably reflecting this inhibition of their secretory trafficking.

Altogether, these observations indicate that cPLA_2_α is the main regulator of transport across the Golgi complex under different conditions of cargo load. GVIIIA-PLA_2_ has a minor role in intra-Golgi transport when cPLA_2_α is normally expressed, but appears to be able to compensate for the lack of cPLA_2_α to support trafficking in cPLA_2_α KO mice.

## Discussion

It has become clear in recent years that cells have at their disposal a vast repertoire of protein-based and lipid-based mechanisms for the bending of their membranes. The former, which have been more extensively characterized, include coat proteins such as clathrin, COPI and COPII complexes, as well as BAR proteins [23],[24],[50], while the lipids include substrates and products of phospholipases, acyltransferases, phospholipid transfer proteins, and flippases [24],[50]. The main finding in this study is that in the Golgi complex, a specific PLA_2_ isoform, namely Group IV cPLA_2_α, is required for the formation of the intercisternal tubules that appear to be involved in intra-Golgi trafficking.

**Figure 10:**
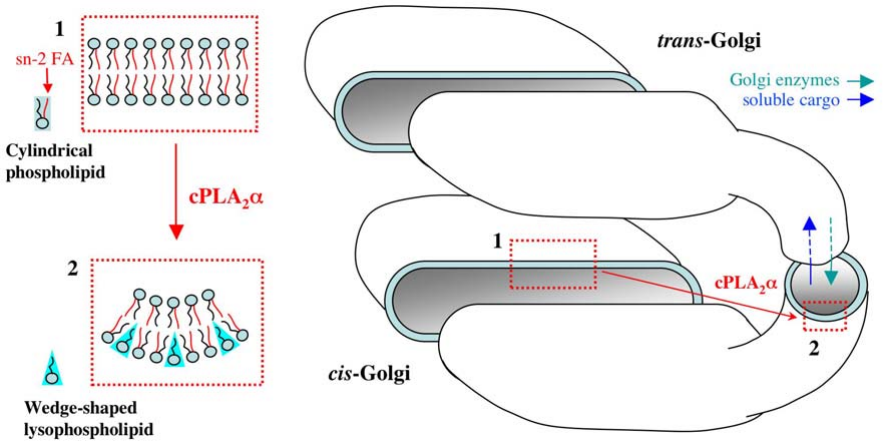
Schematic illustration of cPLA_2_α action at the Golgi complex. Inset 1: cPLA_2_α hydrolyses the fatty acids (FA) at the *sn-2* position of cylindrical phospholipids to form wedge-shaped lysophospholipids. Inset 2: This formation of wedge-shaped lysophospholipids favours generation of spontaneous membrane curvature and transformation of flat cisternae-like membranes into highly curved tubular membranes, which serve as intermediates for transport across the Golgi stack.

The simplest explanation for the role of cPLA_2_α in Golgi tubulation is that this enzyme can induce the rapid accumulation of wedge-like lysolipids at the cisternal rims, resulting in a local increase in spontaneous positive membrane curvature, and hence in tubulation (Figure 10). In addition to curvature, the generation of an intercisternal tubular continuity presumably requires the assembly of the fusion machinery at the tip of the budding tubule, for its connecting with a neighbouring cisterna. This, in turn, is likely to involve the formation of an ARF/COPI coat to recruit these fusion proteins into this bud [18],[63],[64]. Therefore, a simple model that fits our observations is that the cPLA_2_α-generated lysolipids help to create and stabilize the curvature of the necks of COPI buds. This might prevent the fission of such buds into vesicles, allowing these buds to dock and fuse with the next cisternae, creating an intercisternal continuity. It is also possible that the cPLA_2_α-generated lysolipids favour the elongation of buds into tubules, as suggested by the observation that overexpression of cPLA_2_α induces tubulation of the stack structure (Figure 2J). Notably, for these events to occur, diffusion of the lysolipids away from their site of synthesis should be limited by a diffusion barrier at the Golgi rims (perhaps similar to the molecular fences described at the plasma membrane [65]). This fence-like role could involve the COPI coat that resides at the rims of the cisternae. Future studies will elucidate these further components of the tubulation machinery. As noted, the inhibition of cPLA_2_α and the attendant abrogation of the tubules are associated with the arrest of transit through the Golgi complex, indicating that the cPLA_2_α-dependent intra-Golgi tubules are involved in trafficking. Interestingly, a similar association between intra-Golgi tubules and traffic has been reported for the effects of dicumarol. This drug is an activator of the fission-inducing protein CtBP1/BARS, and it suppresses horizontal Golgi tubules (and thus, presumably, also the vertical intercisternal tubules, which, however, were not directly examined) and inhibits intra-Golgi trafficking [66]. Thus, suppressing the tubules appears to result in the arrest of trafficking independently of the molecular mechanisms that underlie tubule disruption.

How do cPLA_2_α-dependent tubules support intra-Golgi trafficking, and which are the tubules—longitudinal or vertical—that are involved in intra-Golgi transport? The simplest hypothesis is that Golgi tubules allow the intercisternal diffusion of molecules crucial for trafficking. Both vertical and longitudinal tubular elements are cPLA_2_α-dependent. The former are much less abundant; however, since traffic requires movement along the *cis*-*trans* (i.e., vertical) axis, they must be functionally crucial (as also supported by investigations in NZ-treated cells). Moreover, while relatively infrequent and difficult to detect [18],[21],[22], vertical tubules have the potential to be a very efficient means of intra-Golgi transit due to the great speed of diffusion over short distances (microns in the Golgi complex). Thus, very few vertical tubules per stack can be sufficient to achieve rapid diffusion between the *cis* and *trans* compartments of the Golgi complex (see Figure S6). This may be the case even when the connections are fewer than those required for complete intra-stack connectivity. For instance, in the Golgi ribbon, where stacks are connected to each other by horizontal membrane bridges, gaps in one stack might be compensated for by connections in neighbouring stacks (in this sense, also horizontal tubules may contribute to *cis*-*trans* diffusion; Figure S6). Connections might be transient; here, again, very few connections need to be present in a stack at any given time to support intra-Golgi diffusion if they rapidly open and close between cisternae (Figure S6). Given the above, the question arises as to the role of intercisternal diffusion in intra-Golgi trafficking of cargo proteins that cross the Golgi by cisternal maturation/progression, such as PC-I and VSVG [35]. The simplest hypothesis is that these tubules allow retrograde movement of Golgi membranes and resident proteins (e.g., enzymes), which is required for maturation [18],[21]. According to this scheme (also discussed elsewhere [18]), during cisternal progression, the Golgi enzymes diffuse through the intercisternal continuities and explore the Golgi space, where they partition according to their physico-chemical properties into those cisternae that have their most favourable composition. This partitioning is driven by a physico-chemical gradient that is maintained across the stack at all times, possibly by the input of compositionally different intermediate compartment membrane into the *cis* cisternae and of endosomal membrane into the *trans*-Golgi. Thus, the arrival of intermediate compartment membranes at the *cis* pole (accompanied by consumption at the *trans*) promotes both enzyme backflow and cisternal progression (above), resulting in the synchronization of these two events and the maintenance of Golgi polarity. Clearly, this model requires more work to fully test it experimentally, but at this stage, it provides a logical explanation of the observations. At the same time, it should be noted that while our data point to a crucial role for tubules, complementary transport mechanisms cannot be excluded. For instance, if Golgi tubules indeed arise from the stabilization of COPI vesicles, as proposed above, it is possible that trafficking might switch between vesicular [67],[68] and connection-mediated modes, with one or the other mechanism prevailing, depending on the cell type and the functional state.

How “general” is the requirement for cPLA_2_α-dependent tubules in trafficking? Our findings show that the role of cPLA_2_α, is very specific for intra-Golgi tubular structures and trafficking. However, other (non-Golgi) tubulation-dependent transport steps have been reported to be blocked by PLA_2_ inhibitors. For instance, ONO (a rather nonspecific inhibitor of many PLA_2_ isoforms) has been shown by us and others to also suppress non-Golgi tubules and to block non-Golgi transport steps that appear to be dependent on tubular intermediates, including endosome-to-plasma-membrane recycling of transferrin [59] and retrograde transport from the Golgi complex to the ER [60]. It is thus possible that these transport steps [59],[60] are regulated by other PLA_2_ isoforms that are located in the different organelles. For instance, cPLA_2_β and cPLA_2_ε have been reported to be located to the early [69] and late [70] endocytic compartments, respectively, and may be involved in the regulation of specific steps of endocytosis that are carried out via tubular carriers and that require PLA_2_ activity [27],[59]. If this is the case, then the PLA_2_ family in general (through different PLA_2_ isoforms), rather than cPLA_2_α itself, could underlie a membrane-bending mechanism based on the induction of spontaneous membrane curvature [24] that is involved in tubulation and trafficking at the different levels of cellular membranes in mammals [27]. A further observation that is most probably related to these considerations is that mice knocked out for cPLA_2_α have reduced fertility, but do not show any other major phenotypes [61],[62], and that secretory transport and Golgi morphology in IMLFs obtained from KO mice [71] are normal. This appears to be because cPLA_2_α in KO cells is functionally replaced by GVIIIA-PLA_2_ (Figure 8). GVIIIA-PLA_2_ also partially localizes at the Golgi complex, where it appears to control tubulation processes (W. Brown, personal communication). Whether the mechanisms of action of this enzyme in trafficking are similar to those of cPLA_2_α is unclear at this time. At the mechanistic level, the properties of GVIIIA-PLA_2_ are not well defined in vivo. Although GVIIIA-PLA_2_ has been shown to have specificity in vitro towards PAF-like lipids, its endogenous substrates remain unknown [72]. Thus, the precise metabolic reactions by which GVIIIA-PLA_2_ supports tubulation remain to be determined.

In conclusion, the activity of cPLA_2_α appears to be an important mechanism for the formation of Golgi tubules in mammalian cells [24]. For other tubulation events (in other organelles or cell types), as noted, this role of cPLA_2_α might be taken on by other PLA_2_ isoforms or even other phospholipases (yeast). Nevertheless, the identification of cPLA_2_α as a player in Golgi tubulation is a key finding, in that it reveals that generation of lysolipids is an important event in the formation of cellular tubules, and it should open the way towards the unravelling of further components of the tubulation machinery. It is now important to elucidate the mode of action of the intercisternal tubular connections and to define their underlying molecular machinery as well as the relationships of these tubules with other key players in intra-Golgi trafficking [73],[74].

## Materials and Methods

### Antibodies and Reagents

Ab sources: Ab against TGN46 from S. Ponnambalam (University of Dundee, Dundee, UK); Ab against PC-I from L.W. Fisher (NIH, Bethesda, MD, USA); Ab against giantin from H-P. Hauri (University of Basel, Basel, Switzerland); Ab against GM130 and ecto-domain of VSVG from M.A. De Matteis (Consorzio Mario Negri Sud, Santa Maria Imbaro, Italy). Ab against GFP and decorin from Abcam (Cambridge, UK); Abs against actin and VSVG from Sigma-Aldrich (Milan, Italy); Abs against cPLA_2_α from Santa Cruz Biotechnology (San Diego, CA, USA) or were produced in our laboratory according to standard protocols. The Alexa 488, 546, and 633 IgG conjugates were from Molecular Probes Europe BV (Leiden, The Netherlands). The NANOGOLD gold-Ab conjugates and the GOLDENHANCE-EM kit were from Nanoprobes (Stony Brook, NY, USA). cDNA sources: cPLA_2_α-GFP from C. Leslie (National Jewish Medical and Research Center, Denver, CO, USA) and T. Hirabayashi and T. Shimizu (University of Tokyo, Tokyo, Japan); cPLA_2_α^(1–522)^ from I. Kudo (Showa University, Tokyo, Japan); C2-GFP from R.L. Williams (MRC, Cambridge, UK); cPLA_2_α^S228C^ from B.P. Kennedy (Department of Biochemistry and Molecular Biology, Merck Frosst Center for Therapeutic Research, P.O. Box 1005, Pointe Claire-DorVal, Canada); L294A αSNAP from R. Burgoyne (University of Liverpool, Liverpool, UK); VSVG-YFP, VSVG-Cherry, VSVG-KDELR, and ARF1-GFP from J. Lippincott-Schwartz (NIH, Bethesda, MD, USA); sialyltransferase-HRP from D.F. Cutler (University College London, London, UK). ONO-RS-082 was from Alexis (Lausen, Switzerland), pyrrophenone was from K. Seno (Shionogi Research Laboratories, Osaka, Japan), pyrrolidine was from Calbiochem (San Diego, CA, USA), bis-BODIPY FL C^11^-PC was from Molecular Probes (Eugene, OR, USA), [^3^H]-AA was from Amersham Pharmacia (Piscataway, NJ, USA).

### Cell Culture, Transfection, RNAi, and Infection with Vesicular Stomatitis Virus

HeLa, MDCK, MC3T3, NRK and PCCL3 cells, HFs, and IMLFs from cPLA_2_α-KO mice were cultured in DMEM (Invitrogen SRL, Milan, Italy) supplemented with 10% foetal calf serum and 1 mM L-glutamine. LipofectAMINE 2000 and Oliogofectamine (Invitrogen, Carlsbad, CA, USA) were used for the cDNA and cPLA_2_α-directed siRNA (SMART Pool, Dharmacon , Chicago, IL, USA) transfections, respectively. Of note, both the mixture of siRNAs as well as two of the individual cPLA_2_α-specific siRNAs from the SMART pool were effective in knocking down cPLA_2_α. The sense oligonucleotide sequences for human cPLA_2_α are:

(#1) GGACAGUCGUUAAGAAGUA,

(#2) GGAGAAACACUAAUUCAUA,

(#3) GGAGAAGACUUUCAGACAA,

(#4) GUACAAGGCUCCAGGUGUU.

The pool of three siRNA (QIAGEN Inc., Valencia, CA, USA) was used to silence mouse cPLA_2_α. The sense oligonucleotide sequences for mouse cPLA_2_α are:

(#1) CCAGATGAATTTGAACGAATA,

(#2) AAGCCTGAGGATTCTCATTTA,

(#3) TAGGAGAAACACTAATTCAAA.

The efficiency of cPLA_2_α knock-down was evaluated by either western blotting or immunofluorescense. Infection of cells with VSV was performed as described previously [18]. For VSVG rescue experiments, HeLa cells were incubated with oligonucleotide #4 (directed against amino acids 649–655) and then transfected with mouse full length cPLA_2_α or the human cPLA_2_α^(1–522)^ mutant. Alternatively, mouse MC3T3 cells silenced for cPLA_2_α with pool of three oligonucleotides were transfected with full length human cPLA_2_α or human cPLA_2_α^(1–522)^ or cPLA_2_α^S228C^ mutants.

### Cell Microinjection

VSV-infected HFs were microinjected with 4 mg/ml anti-cPLA_2_α ab in the presence of FITC or TRITC dextrans during the course of the 40°C block, using an Eppendorf transjector 5246 (Eppendorf, Milan, Italy). They were then shifted to 32°C and processed for confocal microscopy. Similarly, VSV-infected cPLA_2_α-silenced HeLa cells were injected with 2 mg/ml recombinant cPLA_2_α protein in resque experiments.

### Treatment with the αSNAP Mutant

HeLa cells were permeabilized with streptolysin-O and incubated with the recombinant L294A αSNAP (4 mg/ml) protein in the presence of cytosol and an ATP-regenerating system, as described in Kweon et al. [56].

### Immunofluorescence, Confocal Microscopy, and Live-Cell Imaging

For immunofluorescence analyses, the cells were fixed with 4% paraformaldehyde and permeabilised in 0.02% saponin, 0.5% BSA, and 50 mM ammonium chloride prior to their incubation with the primary and secondary antibodies of interest. The cells were mounted in mowiol and examined on a Zeiss LSM 510 META confocal microscope (Carl Zeiss, Gottingen, Germany). All confocal images were obtained using the necessary filter sets for GFP, Alexa 488, and Alexa 546 using a Zeiss Plan-Neofluor 63× oil immersion objective (NA 1.4), with the pinhole set to one Airey unit. Overlap between different markers was quantified using the “Co-localization” module of LSM 3.2 software (Zeiss). Time-lapse images were obtained using a Zeiss LSM510 META confocal microscope. Cells co-expressing GFP and YFP fusion proteins were observed at 32°C, as in the case of VSVG-GFP in 20 mM HEPES buffered DMEM. Temperature was controlled with a Nevtek air stream stage incubator (Burnsville, VA, USA). GFP molecules were excited with the 488 nm line of a krypton-argon laser and imaged using the λ-scan mode of the META detector. Confocal digital images were collected using a Zeiss Plan-Neofluor 63× oil immersion objective (NA 1.4) and GFP and YFP fluorescence was unmixed using LSM 3.2 software. Selective photobleaching in the regions of interest within the cell was carried out on the Zeiss LSM510 using 100 consecutive scans with a 488 nm laser line at full power. Average fluorescence intensities within regions of interests were quantified using the LSM 3.2 software.

### Electron Microscopy

For routine EM and EM tomography, the cells were fixed with 1% glutaraldehyde. EM tomography and morphometry on thin sections and tomograms were all performed as described previously [18]. Vertical intercisternal connections were defined as tubular elements, which link different cisternae within the same stack (or cisternae located at different levels across neighbouring stacks). They show a clearly visible lumen (with minimal diameter of 10 nm at the narrowest point along their length) through at least two subsequent tomogram slices. The number of connections for each stack was calculated in each single tomogram of 200 nm thick single sections and expressed as “connections per stack in section.” For pre-embedding gold labelling, cells were fixed with 4% formaldehyde and 0.1% glutaraldehyde, washed, incubated with the primary ab overnight, and then with Nanogold conjugated Fab fragments of the secondary antibodies (Nanoprobes) for 2 h. The Nanogold particles were developed using the Gold-enhance kit. For visualization of the *trans*-Golgi, NRK cells expressing ST-HRP were fixed as indicated above, washed, and incubated with a mixture of DAB and H_2_O_2_, as previously described [3]. Both HRP and gold-labelled cells were embedded in Epon and sectioned. EM images were acquired from thin sections under a Philips Tecnai-12 electron microscope (Philips, Einhoven, The Netherlands) using an ULTRA VIEW CCD digital camera (Soft Imaging Systems GmbH, Munster, Germany). Quantification of gold particles was carried out using the AnalySIS software (Soft Imaging Systems GmbH, Munster, Germany).

### Endo-H Resistance Assay

To determine Endo-H resistance, cells were initially infected with VSV for 1 h and 32°C. The excess virus was then washed off, and the cells were incubated in DMEM containing 10% HEPES for 2 h at 32°C. The cells were then washed three times with PBS and starved in DMEM without methionine and cysteine for 30 min at 32°C. The cells were then pulsed for 5 min with 200 Ci/ml [^35^S]-methionine in DMEM without methionine and cysteine. To stop the pulse, 10 µl 0.25 m methionine in complete DMEM was added, and the cells incubated for 2 min at 32°C. A subset of the samples was then transferred to ice and this was considered Time 0. Other samples were washed with complete medium and chased for the indicated times at 32°C. At the end of the chase, the cells were washed once in PBS and lysed in 1 ml lysis buffer (70 mM Tris [pH 7.4], 150 mM NaCl, 0.5% SDS, 1% Triton X-100, 1 mM EDTA, and 1 mM PMSF) and incubated for 60–90 min on ice. The lysates were centrifuged, and the supernatants were incubated with an anti-VSVG ab overnight at 4°C. The immune complexes were pulled down using Protein A Sepharose. After washing off the unbound material, the protein was eluted by boiling in Endo-H buffer (0.1 m sodium citrate [pH 5.5], 0.5% SDS, and 1% beta-mercaptoethanol) for 3–4 min. The eluates were then divided into two tubes and one was incubated with 40 U Endo-H overnight. The samples were then boiled in SDS-PAGE sample buffer and resolved on an 8% acrylamide gel, using standard procedures. The gels were then scanned and the percentages of the Endo-H resistant form of VSVG with respect to the total amounts of VSVG were quantified using a FUJIFILM imager or ImageJ software.

### Arachidonic-Acid-Release Assays

For the AA-release assays, HeLa or PCCL3 cells were labelled for 18 h in growth medium with 0.1 µCi/ml [^3^H]-arachidonic acid. The [^3^H]-arachidonic acid released into the medium was quantified in triplicates, as described previously [46]; the radioactivity released from cells is expressed as percentages of the total incorporated radioactivity. [^3^H]-arachidonic acid release was quantified over a period of 15 min in experiments with ionomycin stimulation, and over 3 min intervals in VSVG transport experiments.

### Visualization of PLA_2_ Activity with Fluorogenic Substrate

Visualization of PLA_2_ activity in HeLa cells was performed using 1,2-bis-(4,4-difluoro-5,7- dimethyl-4-bora-3a, 4a-diaza-*s*indacene-3-undecanoyl)-*sn*-glycero-3-phosphocholine, which is known also as bis-BODIPY FL C_11_-PC. Liposomes containing bis-BODIPY FL C_11_-PC were prepared according to the manufacture instructions and incubated with VSV-infected HeLa cells 1 h prior to the release of the 40°C block. The cells were fixed at the end of the 40°C block or at different time intervals after the temperature shift to 32°C; they were then immunolabelled for VSVG and GM130 and examined under confocal microscopy. The intensity of the fluorescent signals derived from bis-BODIPY FL C_11_-PC hydrolysis was quantified in the Golgi area using the LSM 3.2 software.

## Supporting Information

Figure S1 **Inhibition of PLA_2_ activity does not affect the dynamics of Golgi-associated vesicles and the ARF/COP machinery.** (A–E) Control (A, C) and ONO-treated (B, D) HeLa cells were permeabilised with streptolysin O and incubated with rat brain cytosol containing 5 µM ONO (B, D) and the L294A αSNAP mutant (C, D). The permeabilized cells were then fixed and prepared for EM. L294A αSNAP induced the accumulation of vesicles near the stacks in both control (C, arrowheads) and ONO-treated (D, arrowheads) cells. Some stacks were greatly vesiculated (C, D, arrows). (E) Quantification of the round profiles associated with the Golgi stacks in samples treated as indicated in A–D. The PLA_2_ inhibitor ONO did not induce an increase in the number of Golgi-associated vesicles and did not affect the accumulation of Golgi vesicles in cells treated with the L294A αSNAP mutant. (F–H) HeLa cells were transfected with an Arf1-GFP construct and observed under confocal microscopy, and Arf1-GFP was bleached within a small area of the Golgi (outlined by boxes in G, H). The quantification of the GFP signal (F) and the time-lapse images in the absence (G) and the presence (H) of 5 µM ONO reveal a recovery of the fluorescent signal within the bleached areas at the same rate in both control and ONO-treated cells, indicating that the PLA_2_ inhibitor ONO did not affect Arf1 turnover at the Golgi complex. (I–K) HeLa cells were incubated either with 5 µM ONO for 30 min (I) or with 10 µg/ml BFA for 15 min (J); alternatively, they were first treated with 5 µM ONO for 30 min, and then 10 µg/ml BFA was added for an additional 15 min (K). The cells were then fixed and stained for galactosyltransferase (GalT) and β-COP. Alone, the PLA_2_ inhibitor ONO did not affect β-COP association with the Golgi complex (I) and did not prevent BFA-induced displacement of β-COP from the Golgi membranes (compare J and K). Interestingly, BFA treatment did not cause disassembly of the Golgi complex in ONO-treated cells, although β-COP was detached from the Golgi membranes (K). Scale bar: 300 nm (A, C), 350 nm (B, D), 9.8 µm (G, H), 11 µm (I–K).
(0.91 MB JPG)

Figure S2 **Membrane transport in cPLA_2_-siRNAs-treated cells.** (A–D) Control (A, C) and cPLA_2_α-siRNAs-treated (B, D) HeLa cells were transfected with the VSVG-KDELR construct, and incubated at 32°C to allow transport of VSVG-KDELR from the ER to the Golgi complex. The cells were then fixed directly (A, B) or 90 min after an incubation at 40°C (C, D), which trapped the VSVG-KDELR in the ER that had been transported retrogradely from the Golgi complex. Confocal microscopy suggests that VSVG-KDELR was delivered effectively from the Golgi complex to the ER in both control (C) and cPLA_2_α-siRNAs-treated (D) cells. (E, F) Control (E) and cPLA_2_α-siRNAs-treated (F) HeLa cells were kept for 1 h at 4°C with 50 µg/ml transferrin-Cy3, then washed and fixed after a 10 min incubation at 37°C. Confocal images indicate that transferrin uptake is not affected by the cPLA_2_ siRNAs. (G, H) Control (G) and cPLA_2_α-siRNAs-treated (H) HeLa cells were incubated for 30 min at 4°C with 100 µg/ml fluorescent wheat-germ agglutinin (WGA). The cells were then washed and incubated at 37°C for 1 h, and fixed and labelled with an anti-TGN46 ab. In both control and cPLA_2_α-siRNAs-treated cells, WGA was internalized and delivered to the Golgi complex, indicating that the endopathway from the plasma membrane to the endosomes, and from the endosomes to the TGN, are not affected in the cPLA_2_α-siRNAs-treated cells. (I–P) Control (I, K, M, O) and cPLA_2_α-siRNAs-treated HeLa cells (J, L, N, P) were fixed and labelled with anti-mannose-6-phosphate receptor (M6PR) (I, J), anti-EEA1 (K, L), anti-Sec31 (M, N), or anti-β-COP (O, P) antibodies. The patterns of each of these markers were similar in the control and cPLA_2_α-siRNAs-treated cells. Scale bar: 6 µm (A–D), 20 µm (E, F), 60 µm (G–P).
(0.87 MB JPG)

Figure S3 **Inhibition of PLA_2_ activity affects transport through the Golgi complex.** (A–F) HFs (A–D) and NRK cells stably expressing sialyltransferase-HRP (ST-HRP) (E, F) were infected with VSV (A–F), and kept at 40°C for 3 h to accumulate VSVG in the ER, without (A, C, E) and with (B, D, F) 5 µM ONO for the final 15 min of the temperature block. The cells were then fixed directly (A, B) or incubated without (C, E) and with (D, F) 5 µM ONO at 32°C for 45 min before fixing. The fixed cells were double labelled with anti-VSVG and anti-giantin antibodies (A–D), or prepared for immuno-EM using nanogold and the HRP protocol (E, F). After accumulation within the ER (A, B), VSVG was efficiently exported to the plasma membrane in control cells (C) and blocked in the Golgi area in ONO-treated cells (D). EM revealed VSVG in the *trans*-Golgi compartment (E, arrows) labelled with ST-HRP, while in ONO-treated cells most of the VSVG remained within the *cis* portion of the stack (F, arrows), which was negative for ST-HRP. (G–J) HFs were kept at 40°C for 3 h in ascorbate-free medium to accumulate PC-I in the ER (G, H), and treated without and with 5 µM ONO for the last 15 min of the temperature block (I, J). The cells were then incubated without (I) and with (J) 5 µM ONO at 37°C for 45 min in medium with ascorbic acid (to allow PC-I folding and exit from the ER), fixed and double labelled with anti-PC-I and anti-giantin antibodies. While in the control cells PC-I was efficiently exported towards the plasma membrane within transport carriers (I, arrows), ONO treatment induced accumulation of PC-I in the Golgi area (J). (K, L) HFs infected with VSV were metabolically labelled with [^35^S]-methionine and then chased at 32°C in the absence and presence of 5 µM ONO. At the indicated times, the cells were solubilized and digested with endoglycosidase H (Endo-H), which cleaves sugar chains built on the proteins early in the secretory pathway (i.e., before their processing by medial Golgi enzyme mannosidase-II, which convert sugars into endo-H resistant forms). The cell lysates were then separated by SDS-PAGE, and the gels scanned (K). The percentages of the Endo-H resistant form of VSVG with respect to the total amounts of VSVG were quantified (L) using a FUJIFILM imager. The data indicate that VSVG processing to its Endo-H resistant form (which occurs in the medial Golgi) was reduced when PLA_2_ activity was inhibited. (M–O) HFs were infected with VSV, and kept at 40°C for 3 h to accumulate VSVG in the ER. The cells were then incubated in the presence of 5 µM ONO at 32°C for 45 min to allow VSVG exit from the ER. Then the cells were fixed immediately (M) or incubated for an additional 15 min (N) or 45 min (O) in fresh ONO-free medium to allow the recovery of PLA_2_ activity. Immunofluorescent labelling of these ONO-treated cells (M) reveals accumulation of VSVG within isolated Golgi stacks labelled with an anti-giantin ab. Shortly after ONO wash-out (N), partial reconnection of the Golgi stacks into a ribbon (N, arrow) coincided with the reactivation of transport and the appearance of VSVG at the cell surface (N, arrowheads); cells still with disconnected Golgi stacks (N, asterisk) did not show VSVG at the plasma membrane. Complete recovery of PLA_2_ activity resulted in the reassembly of the Golgi stacks into a highly connected structure and the exit of the cargo to the plasma membrane (O). Scale bar: 8.2 µm (A–D, N, O), 300 nm (E, F), 9.8 µm (G–J), 4 µm (M).
(0.77 MB JPG)

Figure S4 **The PLA_2_ inhibitor ONO affects Golgi-associated tubular structures.** (A–G) Control (A, C, E, F) and ONO-treated (B, D, E, G) HFs were fixed for confocal microscopy (A, B) or EM (C–G). (A, C) Confocal images of control cells labelled with an anti-giantin ab and EM show that the Golgi ribbon (A) comprises stacks connected by tubules (C). (B, D) The ribbon is fragmented under 5 µM ONO treatment, producing isolated islands located in a perinuclear area. (E) Morphometric quantification of the surface area of the Golgi complex under EM (mean±SD; *n* = 30 stacks) shows a reduction in tubular profiles in ONO-treated cells. (F, G) Virtual slices from EM tomograms of control cells (see Video S6) show tubular connections between cisternae located at different levels of the stack (F, arrowhead) and a lack of intercisternal connections (see Video S7) in ONO-treated cells (G). (H–J) ONO-treated cells were washed with fresh medium for 15 min, fixed, and prepared for confocal microscopy (H) and EM (I). Cells showed long tubules (H, arrows) that reconnect single Golgi fragments into a ribbon. The EM tomogram (see Video S8) corresponding to the stack shown in (I) provide a three-dimensional model (J), with an intercisternal tubular connection indicated (I, J, arrow). (K) Quantification of vertical connections per stack in sections (mean±s.e.; *n* = 10 stacks) in EM tomograms (see Materials and Methods), in control cells, in ONO-treated cells, and following ONO washout. Scale bar: 7 µm (A, B), 300 nm (C, D, F, G), 3.4 µm (H), 120 nm (I, J).
(0.69 MB JPG)

Figure S5 **VSVG transport in immortalized murine lung fibroblasts from cPLA_2_-KO mice.** Immortalized murine lung fibroblasts from cPLA_2_α KO (IMLFs^−/−^, A–D) and control (IMLFs^+/+^, E–H) mice were infected with VSV. The cells were kept at 40°C for 3 h to accumulate VSVG in the ER. Pyrrophenone (0.5 µm; Pyr; specific cPLA_2_α was added to the cells during the final 15 min of the temperature block (B, D, F, H). The cells were then fixed directly (A, B, E, F) or incubated either without (C, G) or with 0.5 µm pyrrophenone (D, H) at 32°C for 60 min before fixation. The fixed cells were double labelled with anti-VSVG and anti-GM130 antibodies. After accumulation within the ER (A, B, E, F), VSVG was efficiently exported first to the plasma membrane in both untreated (C) and pyrrophenone-treated (D) IMLFs^−/−^ as well as in untreated IMLFs^+/+^ (G). In contrast pyrrophenone-treated IMLFs^+/+^ (H) showed significant amounts of VSVG retained within the Golgi complex. Bar, 7.2 µm (A–H).
(0.45 MB JPG)

Figure S6 **Schematic illustration of how tubular elements can connect cisternae across the Golgi stack.** (A) The scheme shows a portion of a Golgi ribbon, where neighbouring stacks are connected to each other by horizontal membranes bridges (outlined in blue). Lack of vertical connections between some cisternae within one stack can be compensated for by the presence of these connections (outlined in red; 1, 2, 3) in neighbouring stacks. These three vertical connections are sufficient to provide membrane continuum between the *cis* and *trans* compartments across all three of the stacks. (B) This scheme illustrates that only a very few transient connections need to be present in a stack at any given time to support intra-Golgi transport. At Time Point 1, only a single vertical connection (outlined by solid line) is present within the stack and would provide exchange of material between the 2^nd^ and 3^rd^ cisternae. This connection disappears (outlined by dashed line) at Time Point 2; however, the generation of new connections (outlined by solid line) would allow further transport between the 1^st^ and 2^nd^ and 3^rd^ and 4^th^ cisternae. Then the cycle can be repeated.
(0.23 MB JPG)

Video S1 **Arrival of cargo protein to the Golgi complex induces binding of the cPLA_2_α-C_2_ domain to the Golgi membranes.**
(3.64 MB MOV)

Video S2 **EM tomography reveals tubular bridges between cisternae located at the different levels of the Golgi stack in HeLa cells.**
(3.37 MB MOV)

Video S3 **cPLA_2_α siRNAs affect the tubular structures associated with the Golgi stack.**
(2.65 MB MOV)

Video S4 **EM tomography reveals tubular bridges between cisternae located at the different levels of the Golgi stack in NZ-treated HeLa cells.**
(1.06 MB MOV)

Video S5 **cPLA_2_α siRNAs affect the tubular structures associated with the Golgi ministacks in NZ-treated HeLa cells.**
(1.55 MB MOV)

Video S6 **EM tomography reveals tubular bridges between cisternae located at the different levels of the Golgi stack in HFs.**
(4.94 MB MOV)

Video S7 **PLA_2_ inhibition affects the tubular structures associated with the Golgi stack.**
(2.11 MB MOV)

Video S8 **Recovery of PLA_2_ activity induces reassembly of tubular bridges between cisternae located at the different levels of the Golgi stack.**
(2.26 MB MOV)

## Abbreviations

AA: arachidonic-acid
Ab: Antibody
cPLA_2_α: cytosolic PLA_2_
EM: electron microscopy
ER: endoplasmic reticulum
GVIIIA: Group VIIIA
HFs: human fibroblasts
IMLFs: immortalized lung fibroblasts
KDELR: KDEL receptor
KO: knock-out
NZ: nocodazol
PC-I: procollagen-I
PLA_2_: phospholipase A_2_
RNAi: RNA interference
TGN: *trans*-Golgi network
VSV: vesicular somatitis virus
VSVG: vesicular somatitis virus glycoprotein
