# Supplementary figures and images for: Group IV Phospholipase A2α Controls the Formation of Inter-Cisternal Continuities Involved in Intra-Golgi Transport
Source: PLoS Biol. 2009 Sep 15;7(9):e1000194. doi: 10.1371/journal.pbio.1000194 (PMC2732982; doi:10.1371/journal.pbio.1000194)

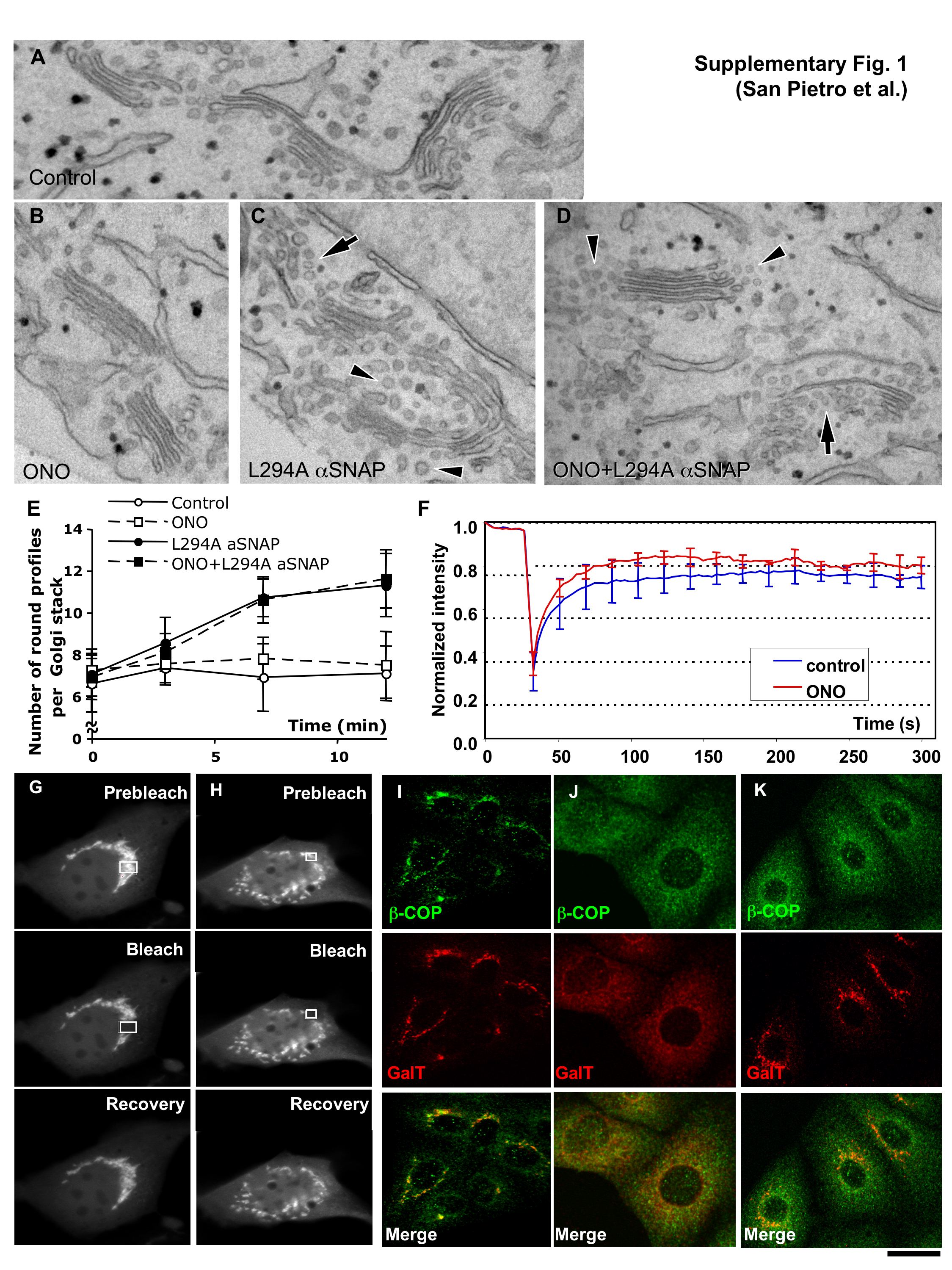

Supplement: Figure S1 — Inhibition of PLA2 activity does not affect the dynamics of Golgi-associated vesicles and the ARF/COP machinery. (A–E) Control (A, C) and ONO-treated (B, D) HeLa cells were permeabilised with streptolysin O and incubated with rat brain cytosol containing 5 µM ONO (B, D) and the L294A αSNAP mutant (C, D). The permeabilized cells were then fixed and prepared for EM. L294A αSNAP induced the accumulation of vesicles near the stacks in both control (C, arrowheads) and ONO-treated (D, arrowheads) cells. Some stacks were greatly vesiculated (C, D, arrows). (E) Quantification of the round profiles associated with the Golgi stacks in samples treated as indicated in A–D. The PLA2 inhibitor ONO did not induce an increase in the number of Golgi-associated vesicles and did not affect the accumulation of Golgi vesicles in cells treated with the L294A αSNAP mutant. (F–H) HeLa cells were transfected with an Arf1-GFP construct and observed under confocal microscopy, and Arf1-GFP was bleached within a small area of the Golgi (outlined by boxes in G, H). The quantification of the GFP signal (F) and the time-lapse images in the absence (G) and the presence (H) of 5 µM ONO reveal a recovery of the fluorescent signal within the bleached areas at the same rate in both control and ONO-treated cells, indicating that the PLA2 inhibitor ONO did not affect Arf1 turnover at the Golgi complex. (I–K) HeLa cells were incubated either with 5 µM ONO for 30 min (I) or with 10 µg/ml BFA for 15 min (J); alternatively, they were first treated with 5 µM ONO for 30 min, and then 10 µg/ml BFA was added for an additional 15 min (K). The cells were then fixed and stained for galactosyltransferase (GalT) and β-COP. Alone, the PLA2 inhibitor ONO did not affect β-COP association with the Golgi complex (I) and did not prevent BFA-induced displacement of β-COP from the Golgi membranes (compare J and K). Interestingly, BFA treatment did not cause disassembly of the Golgi complex in ONO-treated cells, although β-CO [file pbio.1000194.s001.jpg]

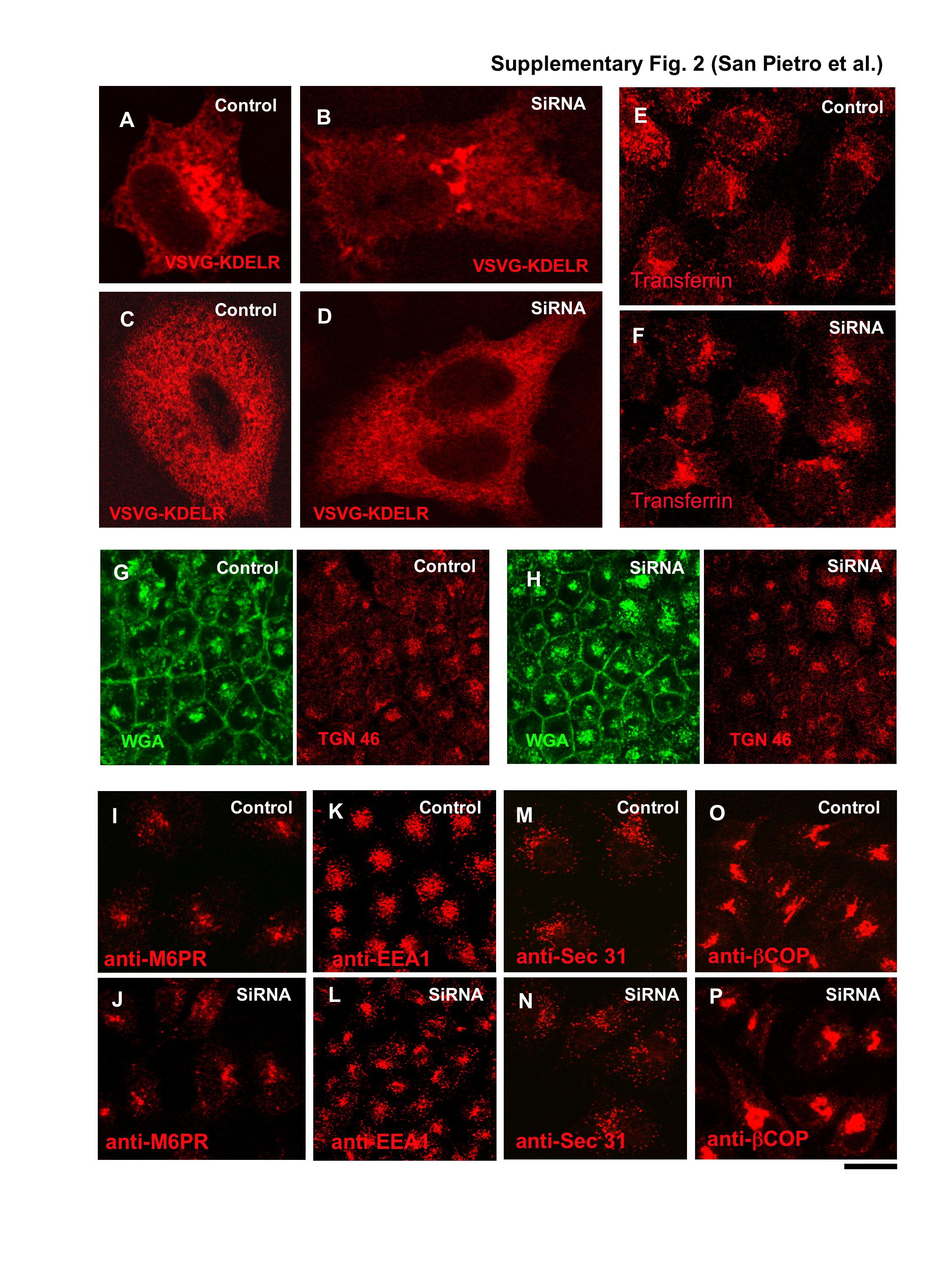

Supplement: Figure S2 — Membrane transport in cPLA2-siRNAs-treated cells. (A–D) Control (A, C) and cPLA2α-siRNAs-treated (B, D) HeLa cells were transfected with the VSVG-KDELR construct, and incubated at 32°C to allow transport of VSVG-KDELR from the ER to the Golgi complex. The cells were then fixed directly (A, B) or 90 min after an incubation at 40°C (C, D), which trapped the VSVG-KDELR in the ER that had been transported retrogradely from the Golgi complex. Confocal microscopy suggests that VSVG-KDELR was delivered effectively from the Golgi complex to the ER in both control (C) and cPLA2α-siRNAs-treated (D) cells. (E, F) Control (E) and cPLA2α-siRNAs-treated (F) HeLa cells were kept for 1 h at 4°C with 50 µg/ml transferrin-Cy3, then washed and fixed after a 10 min incubation at 37°C. Confocal images indicate that transferrin uptake is not affected by the cPLA2 siRNAs. (G, H) Control (G) and cPLA2α-siRNAs-treated (H) HeLa cells were incubated for 30 min at 4°C with 100 µg/ml fluorescent wheat-germ agglutinin (WGA). The cells were then washed and incubated at 37°C for 1 h, and fixed and labelled with an anti-TGN46 ab. In both control and cPLA2α-siRNAs-treated cells, WGA was internalized and delivered to the Golgi complex, indicating that the endopathway from the plasma membrane to the endosomes, and from the endosomes to the TGN, are not affected in the cPLA2α-siRNAs-treated cells. (I–P) Control (I, K, M, O) and cPLA2α-siRNAs-treated HeLa cells (J, L, N, P) were fixed and labelled with anti-mannose-6-phosphate receptor (M6PR) (I, J), anti-EEA1 (K, L), anti-Sec31 (M, N), or anti-β-COP (O, P) antibodies. The patterns of each of these markers were similar in the control and cPLA2α-siRNAs-treated cells. Scale bar: 6 µm (A–D), 20 µm (E, F), 60 µm (G–P). (0.87 MB JPG) [file pbio.1000194.s002.jpg]

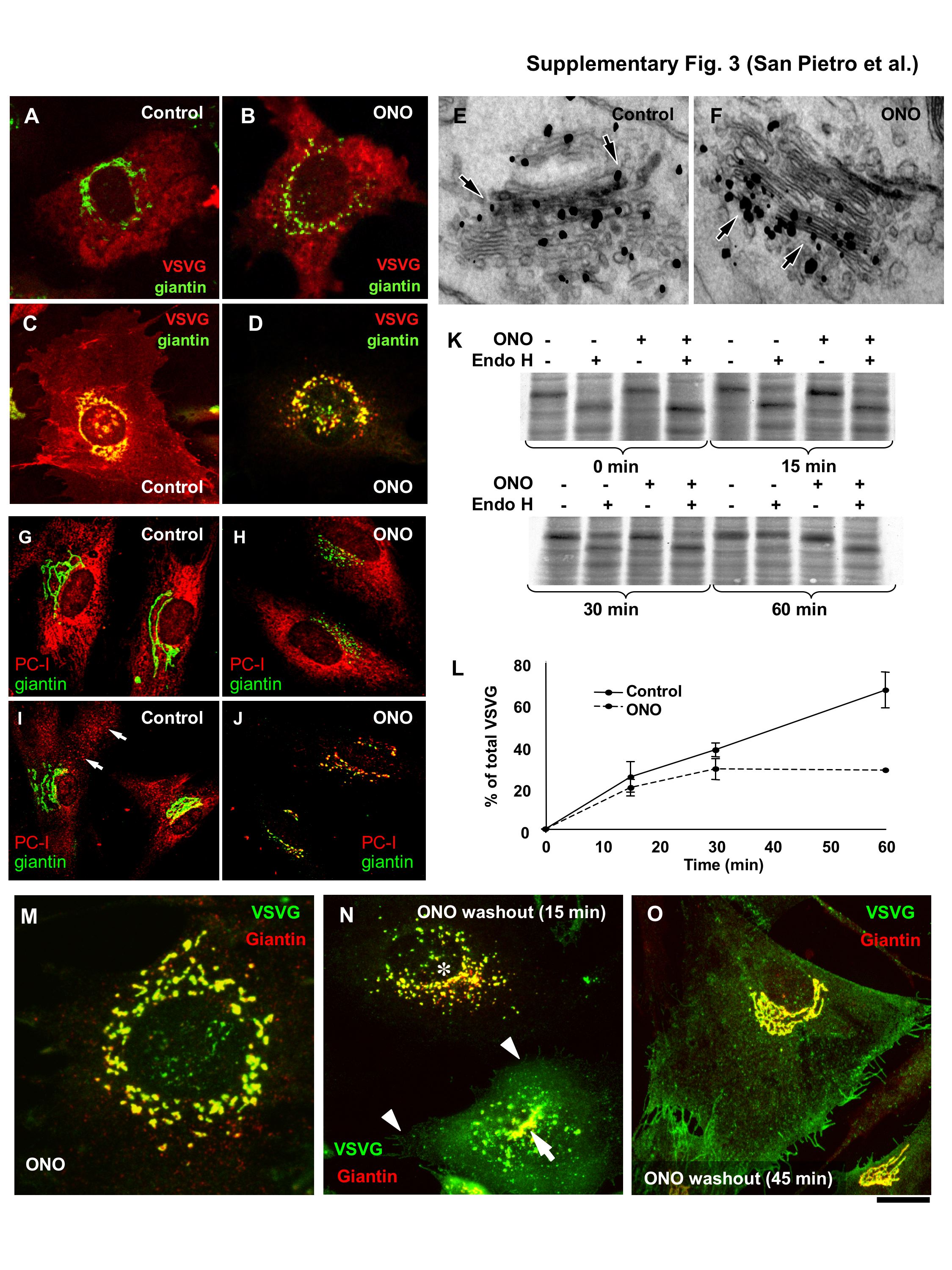

Supplement: Figure S3 — Inhibition of PLA2 activity affects transport through the Golgi complex. (A–F) HFs (A–D) and NRK cells stably expressing sialyltransferase-HRP (ST-HRP) (E, F) were infected with VSV (A–F), and kept at 40°C for 3 h to accumulate VSVG in the ER, without (A, C, E) and with (B, D, F) 5 µM ONO for the final 15 min of the temperature block. The cells were then fixed directly (A, B) or incubated without (C, E) and with (D, F) 5 µM ONO at 32°C for 45 min before fixing. The fixed cells were double labelled with anti-VSVG and anti-giantin antibodies (A–D), or prepared for immuno-EM using nanogold and the HRP protocol (E, F). After accumulation within the ER (A, B), VSVG was efficiently exported to the plasma membrane in control cells (C) and blocked in the Golgi area in ONO-treated cells (D). EM revealed VSVG in the trans-Golgi compartment (E, arrows) labelled with ST-HRP, while in ONO-treated cells most of the VSVG remained within the cis portion of the stack (F, arrows), which was negative for ST-HRP. (G–J) HFs were kept at 40°C for 3 h in ascorbate-free medium to accumulate PC-I in the ER (G, H), and treated without and with 5 µM ONO for the last 15 min of the temperature block (I, J). The cells were then incubated without (I) and with (J) 5 µM ONO at 37°C for 45 min in medium with ascorbic acid (to allow PC-I folding and exit from the ER), fixed and double labelled with anti-PC-I and anti-giantin antibodies. While in the control cells PC-I was efficiently exported towards the plasma membrane within transport carriers (I, arrows), ONO treatment induced accumulation of PC-I in the Golgi area (J). (K, L) HFs infected with VSV were metabolically labelled with [35S]-methionine and then chased at 32°C in the absence and presence of 5 µM ONO. At the indicated times, the cells were solubilized and digested with endoglycosidase H (Endo-H), which cleaves sugar chains built on the proteins early in the secretory pathway (i.e., before their processing by medial Golgi enzyme mannosida [file pbio.1000194.s003.jpg]

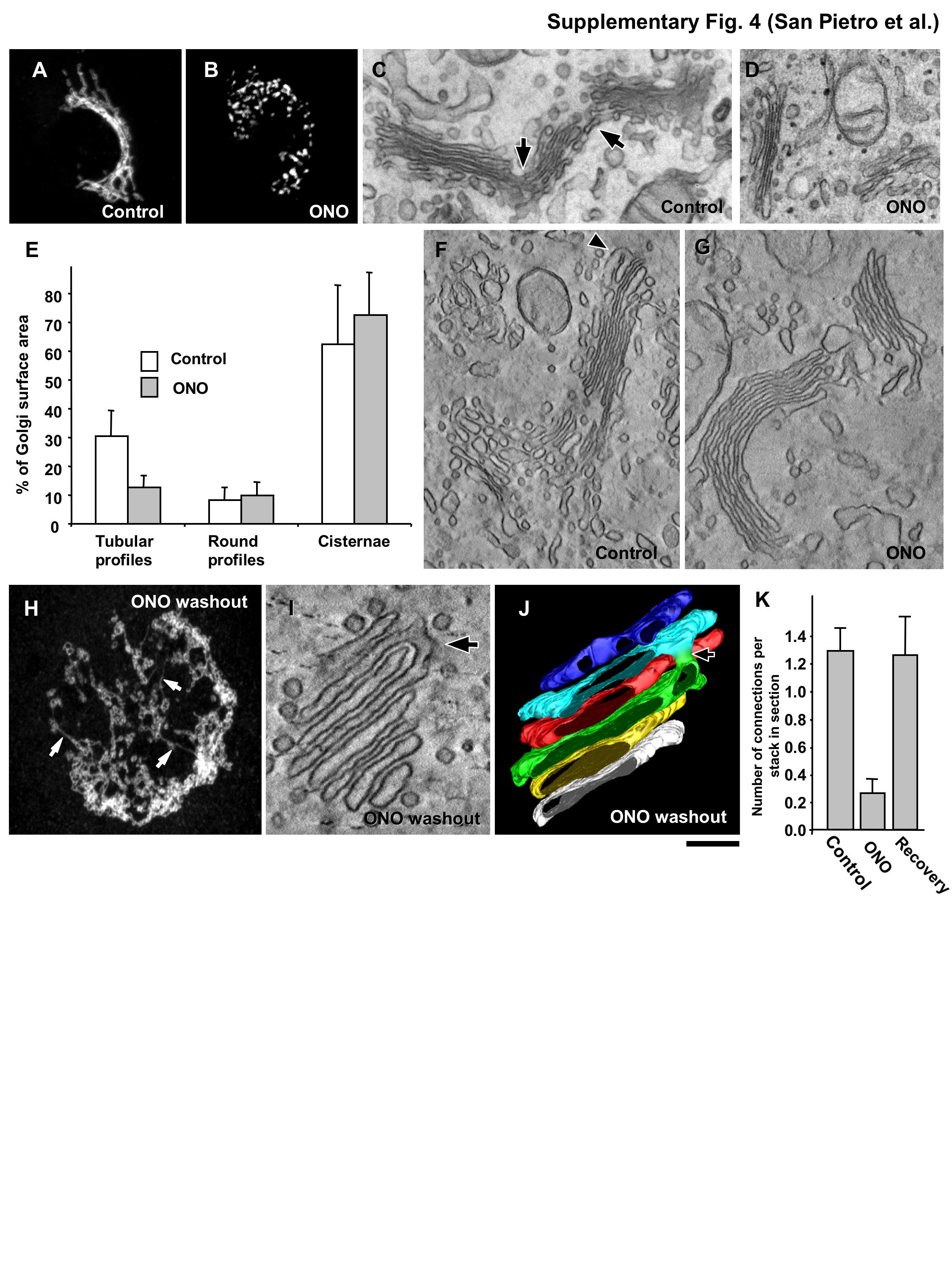

Supplement: Figure S4 — The PLA2 inhibitor ONO affects Golgi-associated tubular structures. (A–G) Control (A, C, E, F) and ONO-treated (B, D, E, G) HFs were fixed for confocal microscopy (A, B) or EM (C–G). (A, C) Confocal images of control cells labelled with an anti-giantin ab and EM show that the Golgi ribbon (A) comprises stacks connected by tubules (C). (B, D) The ribbon is fragmented under 5 µM ONO treatment, producing isolated islands located in a perinuclear area. (E) Morphometric quantification of the surface area of the Golgi complex under EM (mean±SD; n = 30 stacks) shows a reduction in tubular profiles in ONO-treated cells. (F, G) Virtual slices from EM tomograms of control cells (see Video S6) show tubular connections between cisternae located at different levels of the stack (F, arrowhead) and a lack of intercisternal connections (see Video S7) in ONO-treated cells (G). (H–J) ONO-treated cells were washed with fresh medium for 15 min, fixed, and prepared for confocal microscopy (H) and EM (I). Cells showed long tubules (H, arrows) that reconnect single Golgi fragments into a ribbon. The EM tomogram (see Video S8) corresponding to the stack shown in (I) provide a three-dimensional model (J), with an intercisternal tubular connection indicated (I, J, arrow). (K) Quantification of vertical connections per stack in sections (mean±s.e.; n = 10 stacks) in EM tomograms (see Materials and Methods), in control cells, in ONO-treated cells, and following ONO washout. Scale bar: 7 µm (A, B), 300 nm (C, D, F, G), 3.4 µm (H), 120 nm (I, J). (0.69 MB JPG) [file pbio.1000194.s004.jpg]

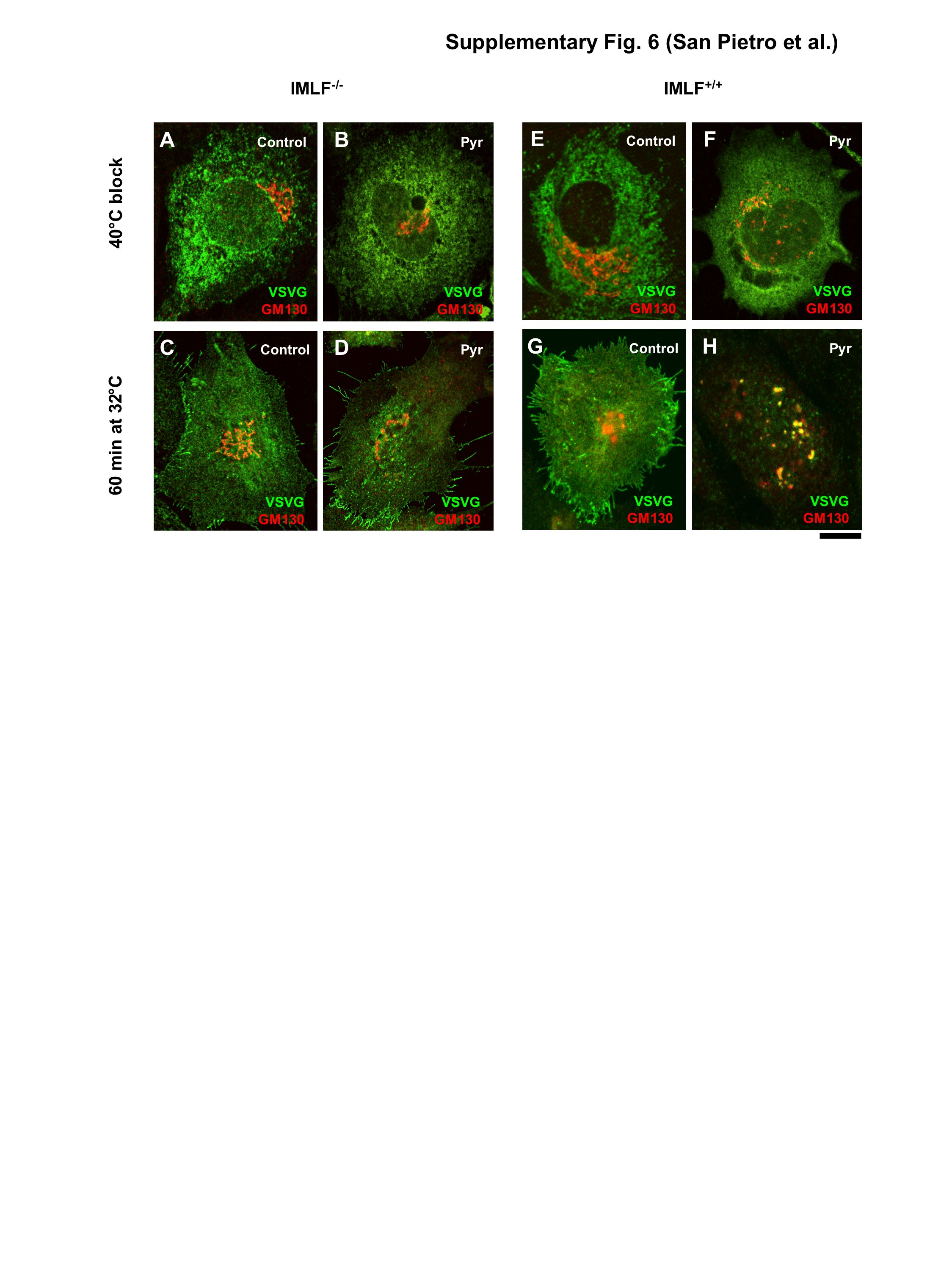

Supplement: Figure S5 — VSVG transport in immortalized murine lung fibroblasts from cPLA2-KO mice. Immortalized murine lung fibroblasts from cPLA2α KO (IMLFs−/−, A–D) and control (IMLFs+/+, E–H) mice were infected with VSV. The cells were kept at 40°C for 3 h to accumulate VSVG in the ER. Pyrrophenone (0.5 µm; Pyr; specific cPLA2α was added to the cells during the final 15 min of the temperature block (B, D, F, H). The cells were then fixed directly (A, B, E, F) or incubated either without (C, G) or with 0.5 µm pyrrophenone (D, H) at 32°C for 60 min before fixation. The fixed cells were double labelled with anti-VSVG and anti-GM130 antibodies. After accumulation within the ER (A, B, E, F), VSVG was efficiently exported first to the plasma membrane in both untreated (C) and pyrrophenone-treated (D) IMLFs−/− as well as in untreated IMLFs+/+ (G). In contrast pyrrophenone-treated IMLFs+/+ (H) showed significant amounts of VSVG retained within the Golgi complex. Bar, 7.2 µm (A–H). (0.45 MB JPG) [file pbio.1000194.s005.jpg]

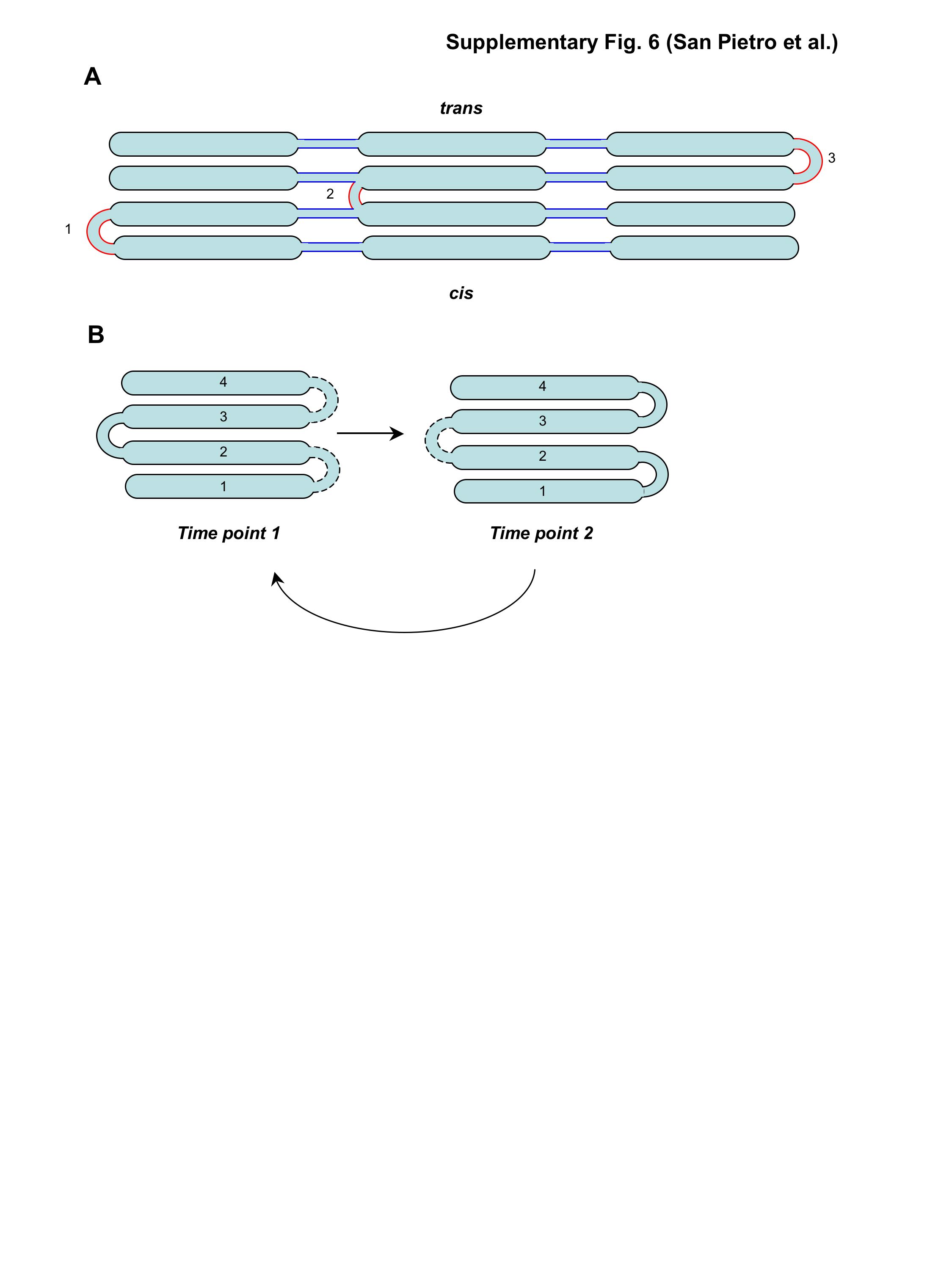

Supplement: Figure S6 — Schematic illustration of how tubular elements can connect cisternae across the Golgi stack. (A) The scheme shows a portion of a Golgi ribbon, where neighbouring stacks are connected to each other by horizontal membranes bridges (outlined in blue). Lack of vertical connections between some cisternae within one stack can be compensated for by the presence of these connections (outlined in red; 1, 2, 3) in neighbouring stacks. These three vertical connections are sufficient to provide membrane continuum between the cis and trans compartments across all three of the stacks. (B) This scheme illustrates that only a very few transient connections need to be present in a stack at any given time to support intra-Golgi transport. At Time Point 1, only a single vertical connection (outlined by solid line) is present within the stack and would provide exchange of material between the 2nd and 3rd cisternae. This connection disappears (outlined by dashed line) at Time Point 2; however, the generation of new connections (outlined by solid line) would allow further transport between the 1st and 2nd and 3rd and 4th cisternae. Then the cycle can be repeated. (0.23 MB JPG) [file pbio.1000194.s006.jpg]
